# Experimental long-term diabetes mellitus alters the transcriptome and biomechanical properties of the rat urinary bladder

**DOI:** 10.1038/s41598-021-94532-7

**Published:** 2021-07-30

**Authors:** Emad A. Hindi, Craig J. Williams, Leo A. H. Zeef, Filipa M. Lopes, Katie Newman, Martha M. M. Davey, Nigel W. Hodson, Emma N. Hilton, Jennifer L. Huang, Karen L. Price, Neil A. Roberts, David A. Long, Adrian S. Woolf, Natalie J. Gardiner

**Affiliations:** 1 School of Medical Sciences, Faculty of Biology, Medicine and Health, University of Manchester, Oxford Road, Manchester, M13 9PT UK; 2 Department of Materials, Faculty of Science and Engineering, University of Manchester, Manchester, UK; 3 Developmental Biology and Cancer Programme, UCL Great Ormond Street Institute of Child Health, London, UK; 4 Royal Manchester Children’s Hospital, Manchester University NHS Foundation Trust, Manchester Academic Health Science Centre, Manchester, UK; 5 Present Address: Department of Anatomy, Faculty of Medicine, King Abdulaziz University, Jeddah, Saudi Arabia

**Keywords:** Atomic force microscopy, Microarray analysis, Bladder, Endocrine system and metabolic diseases, Transcriptomics

## Abstract

Diabetes mellitus (DM) is the leading cause of chronic kidney disease and diabetic nephropathy is widely studied. In contrast, the pathobiology of diabetic urinary bladder disease is less understood despite dysfunctional voiding being common in DM. We hypothesised that diabetic cystopathy has a characteristic molecular signature. We therefore studied bladders of hyperglycaemic and polyuric rats with streptozotocin (STZ)-induced DM. Sixteen weeks after induction of DM, as assessed by RNA arrays, wide-ranging changes of gene expression occurred in DM bladders over and above those induced in bladders of non-hyperglycaemic rats with sucrose-induced polyuria. The altered transcripts included those coding for extracellular matrix regulators and neural molecules. Changes in key genes deregulated in DM rat bladders were also detected in *db*/*db* mouse bladders. In DM rat bladders there was reduced birefringent collagen between detrusor muscle bundles, and atomic force microscopy showed a significant reduction in tissue stiffness; neither change was found in bladders of sucrose-treated rats. Thus, altered extracellular matrix with reduced tissue rigidity may contribute to voiding dysfunction in people with long-term DM. These results serve as an informative stepping stone towards understanding the complex pathobiology of diabetic cystopathy.

## Introduction

Diabetes mellitus (DM) is a leading cause of chronic kidney disease and the pathobiology of diabetic nephropathy has been extensively studied^1^. In contrast, urinary bladder disease associated with DM has been less well investigated. This is despite up to 80% of individuals with DM reporting one or more of: frequency, urgency, or hesitancy of urinary voiding, difficulty in initiating voiding, decreased sensation of bladder fullness and urinary incontinence^2–4^. Indeed, diabetic cystopathy may be under-appreciated because symptoms typically have a gradual onset and can be attributed to other causes (e.g. prostate disease), and most DM clinics do not focus on the bladder. Investigations of affected patients can show increased bladder capacity, impaired detrusor contractility and incomplete voiding^2,4–7^. A urodynamic study of individuals with DM and persistent dysfunctional voiding reported 55% with detrusor smooth muscle (DSM) hypercontractility, 23% with reduced contractility, and 10% with areflexia^8^.

It has been postulated that cystopathy associated with DM is, at least in part, caused by the physical adaptive response to increased urine load combined with metabolic and other direct consequences of DM^9–11^. It would be difficult, however, to determine the possible importance of these factors in DM patients and, critically, there is still a knowledge gap as to the detailed pathobiology of bladder tissues in people with DM. Accordingly, investigators have turned to animal models. The streptozotocin (STZ) rodent model of Type 1 DM has been widely used to study diabetic complications of various organs and tissues, including the urinary bladder^5,11–14^. STZ induces long-lasting hyperglycaemia, polydipsia and polyuria. Moreover, a non-diabetic polyuric state can be induced by the addition of 5% sucrose to rodents’ drinking water, and the effects of this can be compared with STZ-induced DM. Using these approaches, a temporal evolution in bladder dysfunction in experimental DM has been described, starting with a hyperactive compensated bladder leading over several months to a hypoactive decompensated bladder^7,12,14,15^. It was found that such urodynamic abnormalities could be prevented, or partially reversed, by insulin treatment^9,10^. Moreover, functional changes in STZ-diabetic rodents at early time points resembled those seen in sucrose-administered animals^9^. At later time points, however, differences in bladder function emerged between these experimental groups with decreased peak voiding pressure and incomplete voiding observed from 12 weeks in STZ-diabetic but not in sucrose-administered mice^9^.

Previous studies have characterised biochemical, including metabolomic^16^, transcriptomic^17,18^ and protein^19^ changes in bladders 1 and 8 weeks-post STZ. We hypothesised that long-term diabetic cystopathy would have a characteristic molecular signature generated by the combination of sustained polyuria and the metabolic consequences of DM itself. To our knowledge, head-to-head comparisons of the molecular impact of longer duration DM with polyuria per se have not been reported. Sixteen weeks after induction of DM, as assessed by RNA arrays, wide-ranging changes of gene expression occurred in DM bladders over and above those induced in non-hyperglycaemic rats with sucrose-induced polyuria. The altered transcripts included those coding for extracellular matrix (ECM) regulators and neural molecules. Prompted by our bioinformatics results, we examined bladder wall collagen by histology and, for the first time, described altered nanomechanical properties of diabetic rat bladders using state-of-the-art atomic force microscopy (AFM). Finally, we measured expression of specific genes in *db*/*db* mouse bladders, noting that similar transcriptional aberrations occurred in a Type 2 DM model.

## Methods

### Animal studies

Experiments were conducted using adult male Wistar rats (start weight 300–400 g; Charles River, UK) and *db*/*db* and *db*/*lean* (control) adult male mice on a BKS background (Envigo, Blackthorn, UK) in accordance with the UK Animals (Scientific Procedures) Act 1986, EU-201063 (PPL: 7007844; PE52D8C09) and institutional ethical approvals (University of Manchester Animal Welfare and Ethical Review Board, and University College London Local Ethics Committee) in compliance with the ARRIVE guidelines.

Rats were randomly allocated into treatment groups: age-matched control, diabetic or sucrose-treated groups. Diabetes was induced with an intraperitoneal injection of STZ (55 mg/kg in sterile saline) administered after overnight fasting. Glucose levels in tail vein blood were measured 3 days post-STZ using an Accu-chek Aviva Blood Glucose Meter to confirm hyperglycaemia (> 15 mmol/L). Rats were group-housed (2–3 per cage) in individually ventilated cages (Double-decker cage, Techniplast, UK) under a 12:12 h light:dark cycle, with standard laboratory chow (Special Diet Services, UK) and water provided ad libitum, checked daily and weighed regularly as previously described^20^. Slow-release insulin (half-pellet delivering ~ 1U insulin/day; Linshin, Canada) were implanted subcutaneously (under isoflurane anaesthesia) to diabetic rats at 12 weeks post-STZ to prevent morbidity (if rats showed loss of condition, they received this insulin implant earlier). Sucrose-treated rats received 5% sucrose in their drinking water. Controls were age-matched untreated rats. Fluid intake was monitored per cage every 2 weeks by weighing water bottles over two consecutive days to calculate mean daily intake per rat. At the final timepoint, water consumption was measured in individual rats separated from their cagemates using a ‘buddy-barrier’ to reduce isolation stress. Urine volumes were not assessed in this study. A glucose tolerance test of fasted sucrose-treated and age-matched control rats was performed at 15 weeks. A blood sample was taken from the tail vein (time 0), then a 20% glucose solution (2 g/kg body weight) administered by oral gavage. Blood samples (~ 20 μl) were collected from the tail vein at 15, 40, 60, and 120 min post-gavage.

### Tissue harvest and analysis

After 16 weeks, rats were terminally-anaesthetised with isoflurane, culled by decapitation and a sample of core blood collected into lithium heparin tubes (Greiner), centrifuged and supernatant stored at − 80 °C. Body composition of cadavers was assessed by EchoMRI (Echo Medical Systems) to determine proportions of fat and lean mass. Urinary bladders were dissected, emptied of urine by puncture with a hypodermic needle, weighed, then either snap frozen on dry ice and stored at − 80 °C, or fixed for 4 h in 4% paraformaldehyde (in 0.1 M phosphate buffer, pH 7.4, on wet ice). Fixed bladders were rinsed in PBS and transferred to 70% ethanol until processing into parrafin blocks. 5 μm transverse sections through the equatorial region of the bladder were cut and mounted onto glass slides and stained with haematoxylin and eosin (H&E) using an Leica Autostainer XL (Leica, UK) or in 1% picrosirius red (PSR) solution (diluted in 1.3% picric acid; 1 h) followed by clearing in 0.5% glacial acetic acid. Images from stained sections were collected using a 3D-Histech Panoramic-250 Microscope Flash Slide Scanner and Panoramic Viewer (3DHistech Ltd., Hungary). Wall thickness, relative areas of bladder DSM and number of nuclei in DSM (from 6 randomly selected fields of view in the DSM, × 40 magnification) were measured using Panoramic Viewer software (from 3 to 4 sections per animal, each section > 50 μm apart) and means used to calculate group means. Collagen expression was measured using ImageJ and expressed as percentage of PSR staining per tissue area (for DSM and lamina propria layers). In addition, PSR was visualised under cross-polarised light, to examine expression of birefringent collagen, and alignment of the collagen fibrils (coherency) using an Image J plugin (OrientationJ;^21^. Analysis was conducted in a blinded fashion from 4 fields of view in 3 tissue sections per rat and group means obtained for each treatment group.

### Assays

Fasting and non-fasting serum insulin levels of control and sucrose-treated rats were assessed using the rat insulin ELISA (Mercodia, Sweden, catalogue No. 10-1250-01). Total cholesterol and triglyceride levels were determined using Cholesterol Fluorometric Assay and Triglyceride Colorimetric Assay kits (Cayman Chemical, USA). Mice were fasted for 5 h prior to euthanasia when blood was collected and glucose levels assayed in the serum using a colorimetric assay (Cayman Chemicals, USA). Albumin in mouse urine was measured by ELISA (Bethyl Laboratories, USA).

### RNA microarrays, RT-qPCR and analysis

Urinary bladder RNA was isolated from hemisected (sagittal) rat urinary bladders using Trizol (Ambion, USA). The quality and quantity of extracted RNA was measured, using a NanoDrop 2000 spectrophotometer. Fragmentation, labelling (Affymetrix Genechip WT Terminal labelling kit, Affymetrix, UK) and subsequent hybridization was performed using Affymetrix GeneChip Rat Genome 230 2.0 Arrays. dChip (V2005) was used to perform outlier analysis and check the technical quality control^22^ and data was analysed (Study 1: control *n* = 3; diabetic *n* = 4*;* Study 2: control *n* = 3, diabetic *n* = 5 and sucrose-treated *n* = 4). Gene expression analysis quantile normalisation, and background correction were conducted using RMA in Bioconductor^23^ and differential expression was done with limma in Bioconductor (PMID: 15461798). Gene lists of differentially expressed genes were controlled for false discovery rate (fdr) errors using the method of q-value^24^. Principal component analysis (PCA) was performed with Partek Genomics Suite (Partek Inc., USA.), Suppl. Fig. 1. Transcriptomic changes and pathway enrichment were assessed using Ingenuity Pathway Analysis [IPA; Qiagen (www.qiagen.com/ingenuity)] software. For cluster analysis, differential gene expression was filtered by *p* value < 0.05 and fold change >  ± 1.3 in any of the three comparisons (control vs diabetic, control vs sucrose-treated, diabetic vs sucrose-treated). This genelist was segregated into clusters based on similarity of expression profile across the 3 experimental conditions using a κ-means clustering algorithm (clustering on the means (log 2 and z-transformed) using Manhattan Distance with the "Super Grouper" plugin of maxdView software (available from http://bioinf.man.ac.uk/microarray/maxd/). Clustering results were visualized using MultiExperiment Viewer (version 4.8.1). Cluster lists were subsequently analysed by STRING (https://string-db.org/ Version 10.5) to assess known interactions and pathway enrichments.

To validate array results, quantitative polymerase chain reaction (QPCR) TaqMan Gene Expression assays were undertaken for selected transcripts, the assay plate was placed in the StepOne Real Time PCR‐system (Applied Biosystems). Cycling conditions: 50 °C for 2 min followed by 95 °C for 10 min, 95 °C for 15 s, followed by 60 °C for 1 min for 40 cycles. StepOne Software v2.3, (Applied Biosystems) was used to analyse results relative to the housekeeping transcript *Actb*. In addition, RNA was isolated from bladders of 16-week old *db*/*db* and *db*/*lean* mice. 500 ng of RNA was used to prepare cDNA (iScript cDNA synthesis kit, Biorad, UK). qRT-PCR was performed in duplicate, using *Hprt* as a housekeeping gene, as previously described^25^. All primer details are shown in Suppl. Table 1.

### Atomic force microscopy

Detrusor muscle nanomechanics were tested using atomic force microscopy (AFM). Fresh-frozen bladder tissue embedded in OCT compound was sectioned at 10 μm (Leica CM3050S cryostat, Leica, Wetzlar, Germany) and thaw-mounted onto glass slides. OCT was removed by washing 3 times with double distilled water and the slides were left to air-dry for 8–12 h, then stored at 4 °C until analysis by AFM within 1–2 days.

AFM was performed using a JPK Nanowizard 4 Atomic Force Microscope (Bruker, Coventry, UK). The indentations were performed inside a droplet of 0.22 μm filtered, double distilled water using a spherical probe (approx. 5 μm diam.) attached to a cantilever with a nominal spring constant of 2.8 N/m (CP-FM-BSG; sQube, Bickenback, Germany); absolute cantilever spring constants were determined before each session using the JPK non-contact calibration method. Five separate 10 μm^2^ regions of detrusor muscle were measured (control *n* = 4; diabetic *n* = 4*;* sucrose-treated *n* = 4), and each area was tested in a grid pattern of 100 sampling points with the following indentation parameters: setpoint = 50 nN, z length = 2.0 μm, extend speed = 1.0 μm/s, and retract delay = 500 ms.

Determination of Young’s modulus (by application of the Hertzian model) was performed using the JPK Data Processing analysis package. The Poisson’s ratio for the sample was assumed to be 0.25. The data were batch processed by area to determine a mean modulus and standard deviation for each set of 100 indents. The reported mean and standard deviation were recorded after any curves with values above or below two standard deviations from the mean were removed from the dataset in order to account for failed indentations.

### Statistical analysis

Graphs are expressed as scatterplots of the data from individual rats and show group mean ± standard deviation, or median (± interquartile range) as appropriate for distribution of datasets. GraphPad Prism 7.0 was used for statistical analysis (unpaired t-tests, one-way or two-way ANOVA followed by Tukey’s post hoc test or Kruskal–Wallis followed by Dunn’s multiple comparison test as appropriate for the datasets). A *p* value < 0.05 was considered statistically significant.

## Results

### Bladder hypertrophy occurs in STZ-diabetic and in sucrose-treated polyuric rats

**Figure 1:**
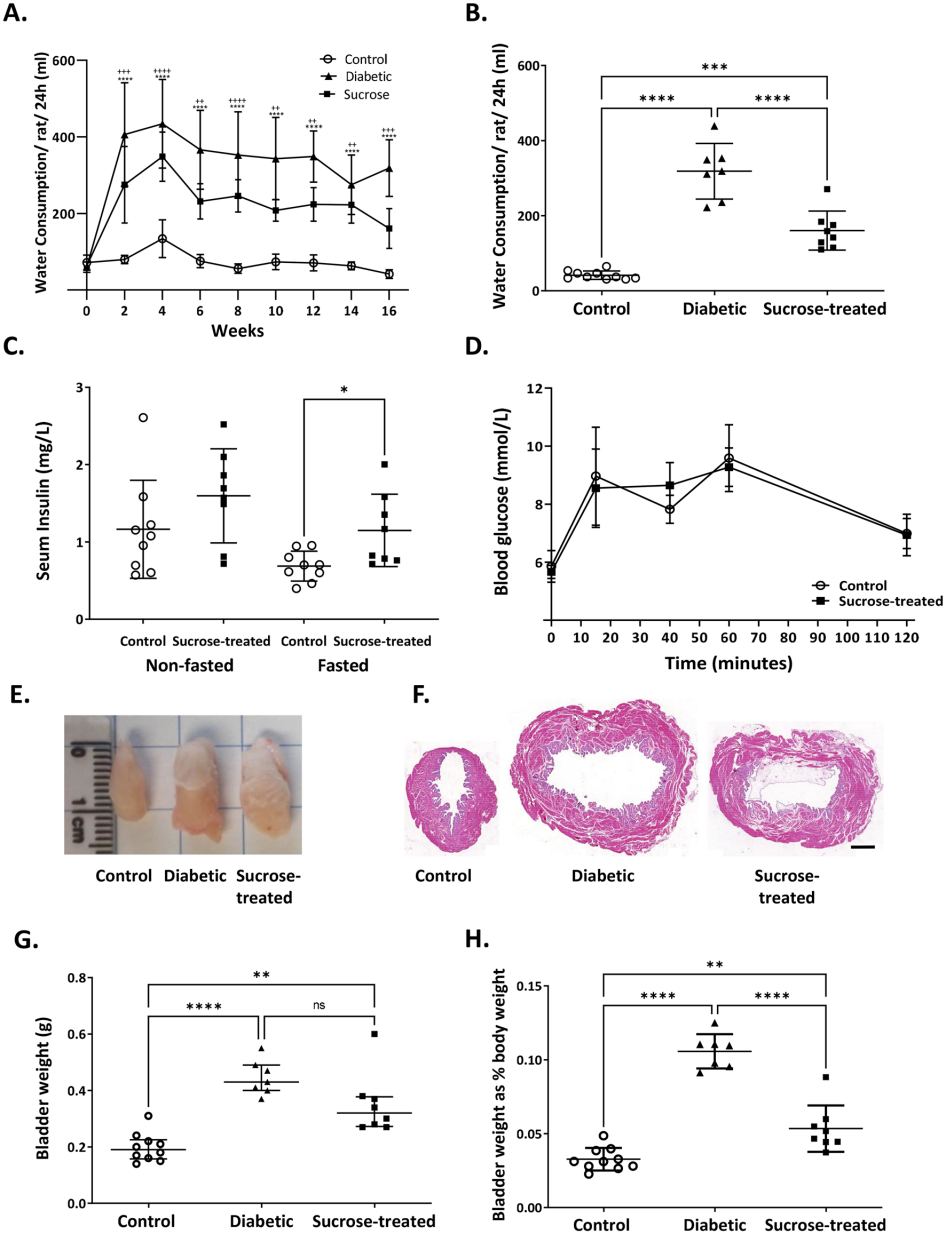
Both diabetic and sucrose-treated rats develop polydipsia and bladder hypertrophy. (**A**) Fluid intake was monitored per cage every 2 weeks, by weighing water bottles over two consecutive days to calculate mean daily intake per rat. Both diabetic rats and sucrose-treated rats drank significantly more than age-matched controls at all timepoints. Diabetic rats drank more than sucrose-treated rats at the majority of time points tested (except 4 and 14 weeks, *p* > 0.05) * denotes diabetic group versus control and ^+^ denotes sucrose-treated group versus control; two way ANOVA and Tukey’s post hoc test: ^##/^**p < 0.01; ^###/^***p < 0.001; ^####^/****p < 0.0001). (**B**) At the final 16 week timepoint, water consumption was measured in individual rats, by separation of cagemates with a ‘buddy barrier’ in their home cage (to minimise isolation stress). Diabetic rats (*n* = 7) and sucrose-treated rats (*n* = 8) drank significantly more than controls (*n* = 10), and diabetic rats drank more than the sucrose-treated rats (one way ANOVA followed by Tukey’s post hoc test, ***p < 0.001****p < 0.0001). (**C**) Insulin levels were not significantly different in sucrose-treated rats compared to control rats in non-fasted conditions (*p* > 0.05), fasted serum insulin levels were increased compared with control rats (*p = 0.016, t-test). (**D**) However, an oral glucose tolerance test conducted at 15 weeks revealed no significant differences between control and sucrose-treated rats (*p* > 0.05, two-way repeated measures ANOVA followed by Tukey’s post-hoc test). Data (**A**–**D**) are expressed as mean ± SD. The empty urinary bladders of diabetic and sucrose-treated rats appeared larger (**E**, **F**) and were heavier (**G**) than controls with no significant difference in bladder weights between the diabetic and sucrose-treated rats. Bladder weight expressed as proportion of body weight (**H**) highlighted the increase in bladder size despite the diabetes-associated body weight deficit. Data are expressed as (**G**) median ± interquartile range, analysed by Kruskal–Wallis followed by Dunn’s multiple comparison test or (**H**) mean ± SD analysed by one way ANOVA followed by Tukey’s post hoc test; (*p < 0.05; **p < 0.01; ****p < 0.0001; ns *p* > 0.05).

To distinguish the impact of DM from increased fluid intake on the bladder, adult male Wistar rats with STZ-induced DM (‘Diabetic’) were compared with age-matched untreated rats (‘Control’) and with rats administered 5% sucrose in their drinking water (‘Sucrose-treated’). Both diabetic and sucrose-treated rats drank significantly more than controls throughout the study and diabetic rats drank significantly more than sucrose-treated rats at most time points (Fig. 1A) including the 16-week endpoint (Fig. 1B). Diabetic rats were hyperglycaemic and weighed less (with altered body composition (lean and fat mass)) than control or sucrose-treated rats at the end of the study (Table 1). Sucrose-treated rats were not hyperglycaemic nor significantly different in body weight or composition than controls (Table 1). Neither the diabetic rats nor the sucrose-treated rats had significant differences in blood cholesterol or triglycerides compared with the controls at this timepoint. To determine whether sucrose-treated rats had developed Type 2 DM, insulin levels were measured and a glucose tolerance test was performed on fasted control and sucrose-treated rats. In the non-fasted state, control and sucrose-treated rats had similar levels of serum insulin; in the fasted state sucrose-treated rats showed elevated levels of insulin compared with controls (Fig. 1C) but this was not associated with impaired glucose tolerance (Fig. 1D).

**Table 1:** Metabolic indices: STZ-diabetic rats developed hyperglycaemia and were significantly lighter than untreated rats 16 weeks post-STZ.

Experimental group *(n number)*	Start weight (g)	Terminal measurement (16 weeks)					
		End weight (g)	Fat mass (% body weight)	Lean mass (% body weight)	Blood glucose (mmol/l)	Cholesterol (mg/dl)	Triglyceride (mg/dl)
Control untreated *(10)*	341 ± 40	606 ± 70	15.0 ± 6.0	74.1 ± 5.1	6.3 ± 0.9	126.6 ± 30.1	113.8 ± 32.7
STZ-diabetic *(7)*	354 ± 23	422 ± 41****	5.8 ± 1.1**	82.0 ± 2.8*	30.6 ± 5.3****	145.9 ± 50.1	159 ± 85.1
Sucrose-treated *(8)*	366 ± 12	657 ± 10 ^++++^	16.3 ± 5.1 ^++++^	72.8 ± 5.1 ^+++^	6.6 ± 0.8 ^++++^	113.2 ± 29.5	149.4 ± 45.5

The sucrose-treated group did not develop hyperglycaemia or hyperlipidaemia and were not significantly different in body weight or composition (% fat/lean mass, assessed by Echo MRI) from the untreated control rats after 16 weeks. Data represent mean ± standard deviation. Statistical analysis was conducted using one-way ANOVA followed by Tukey’s post hoc test (*) denotes the comparison with the untreated control group and (+) denotes the comparison with diabetic rats (*p < 0.05, **p < 0.01; ^+++^*p* < 0.001: ****/^++++^*p* < 0.0001).

The increased drinking and, by implication, consequent increased urine output, was associated with enlarged urinary bladders (Fig. 1E shows excised post-mortem bladders drained of urine). On gross inspection both diabetic and sucrose-treated rats had thicker bladder walls and enlarged lumens compared with controls (Fig. 1F). Both diabetic and sucrose-treated rats had significantly heavier bladders than controls with no significant difference between diabetic and sucrose-treated groups (Fig. 1G: control: 0.19 g (IQR = 0.23–0.16); diabetic: 0.43 g (IQR = 0.49–0.4); sucrose-treated: 0.32 g (IQR = 0.38–0.27). Bladder weight factored for body weight (Fig. 1H) highlighted the increase in bladder size occurred despite the diabetes-associated deficit in body weight (Table 1).

**Figure 2:**
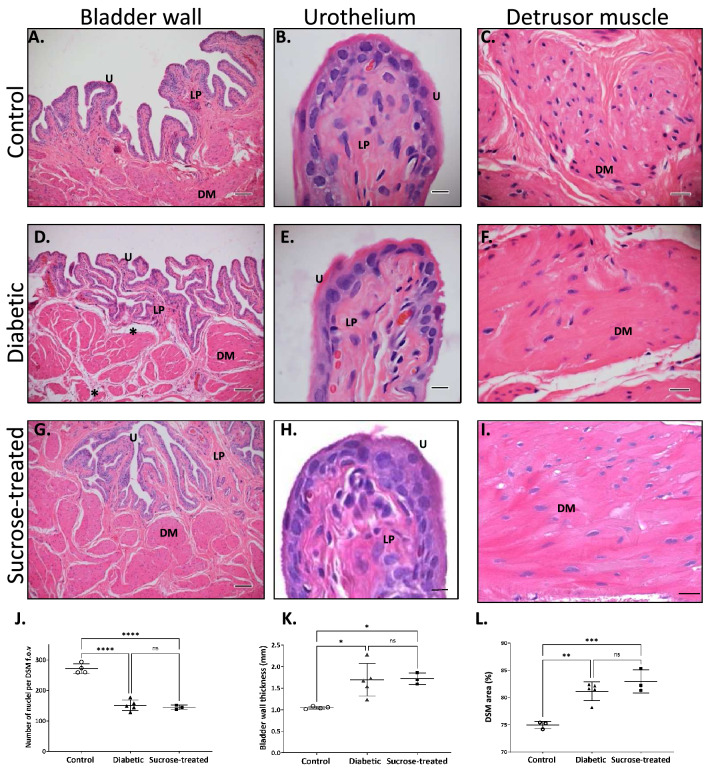
Histological characterisation of the urinary bladder of control, diabetic and sucrose-treated rats. Representative hematoxylin and eosin stained transverse sections taken from the equatorial region of urinary bladders from (**A**–**C**) control, (**D**–**F**) diabetic and (**G**–**I**) sucrose-treated rats after 16 weeks. Hypertrophy occurred in DSM (**F**, **I**) of both diabetic (*n* = 5) and sucrose-treated rats (*n* = 3), with fewer visible nuclei per region of DSM (**J**), thickening of the bladder wall (**K**) compared to controls (*n* = 4). The relative area occupancy of DSM in the bladder wall increased similarly in both diabetic and sucrose-treated groups (**L**). Expanded unstained regions around DSM bundles of diabetic rats are also noted (asterisks, **D**). u = urothelium; LP = lamina propria; DM = detrusor muscle; Scale bars (**A**, **D**, and **G**) = 100 μm; (**B**, **E**, and **H**) = 10 μm; (**C**, **F**, and **I**) = 20 μm. Data are expressed mean ± SD analysed by one way ANOVA followed by Tukey’s post hoc test (*p < 0.05; ****p < 0.0001).

Histological examination (Fig. 2A–I) revealed detrusor (Fig. 2C, F, I), but not urothelial (Fig. 2B, E, H), hypertrophy, with a decreased number of nuclei by area in DSM, in both diabetic and sucrose-treated rats (Fig. 2J) and thicker bladder walls (Fig. 2K: control: 1.1 ± 0.03 mm; diabetic: 1.7 ± 0.4 mm; sucrose-treated: 1.7 ± 0.1 mm) but no significant difference between these two experimental groups. The relative area occupancy of DSM in the bladder wall increased similarly in both diabetic and sucrose-treated groups (Fig. 2L).

Thus, STZ-induced diabetic and sucrose-treated rats had similar increases of bladder weight and DSM hypertrophy. Since the sucrose-treated rats had not developed hyperglycaemia or impaired glucose tolerance by 16 weeks, they were an appropriate experimental group with which to study the potential molecular impacts of polyuria itself without a diabetic milieu.

### Transcriptomic aberrations in bladders of rats with DM or non-diabetic polyuria

**Figure 3:**
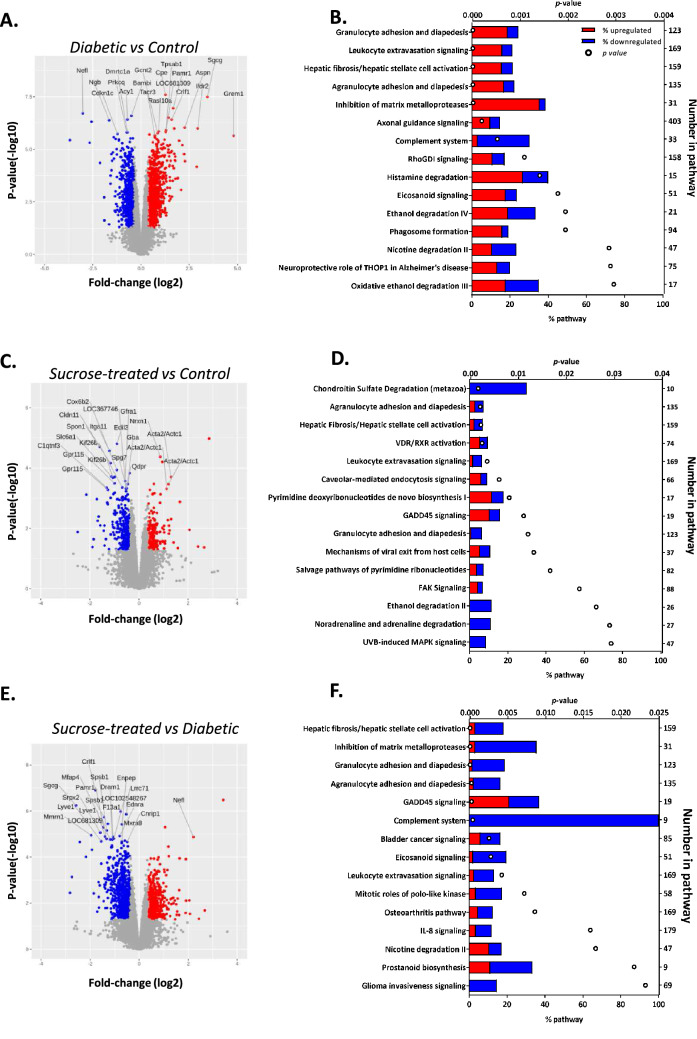
Ingenuity pathway analysis revealed differences in overrepresented pathways in the remodelled bladder of diabetic and sucrose-treated rats. RNA was isolated from hemisected (sagittal) rat urinary bladders (control *n* = 6; diabetic *n* = 9; sucrose-treated *n* = 4) and processed through Affymetrix GeneChip Rat Genome 230 2.0 Arrays. dChip, gene expression analysis quantile normalisation, background correction and differential expression were performed. Volcano plots show dysregulated transcripts arranged by log_2_-fold change and *p*-value (Red: upregulated ≥  ± 1.3 fold change and *p* < 0.05; Blue: downregulated ≥  ± 1.3 fold change and *p* < 0.05; Grey: other transcripts) in (**A**) diabetic versus control (**C**) sucrose-treated versus control and (**E**) sucrose-treated versus diabetic comparisons. The most significantly altered transcripts (with known gene symbols) are annotated. (**B**, **D**, **F**) Bioinformatics analysis using Ingenuity Pathway Analysis (Qiagen) identified overrepresented canonical pathways for each comparison, which are organised by pathway names (left y-axis) and significance (*p*-value, top x-axis); the bars show the % of differentially expressed transcripts (bottom x-axis; Red: upregulated; Blue: Downregulated). For information, the total number of molecules ascribed to each canonical pathway is shown on right-y-axis. For clarity, only the fifteen most significantly overrepresented pathways in each comparison are shown (for all overrepresented pathways see Suppl. Figs. 4, 5, and 6).

GeneChip Rat Genome 230 2.0 Arrays were used to comprehensively characterise and compare transcriptomic changes between control, diabetic and sucrose-treated rats. The most significantly-altered transcripts (with known genes) in the three comparisons (diabetic vs control; sucrose-treated Vs control; sucrose-treated vs diabetic) are depicted in Fig. 3A, C, E, and the largest fold-changes (FC; *p* < 0.05) listed in Suppl. Tables 2, 3, and 4. A total of 1467 transcripts were differentially expressed in diabetic bladders versus controls (834 upregulated and 633 downregulated; *p* < 0.05) (Fig. 3A). Suppl. Table 2 highlights the ten transcripts with greatest fold change (FC) in this comparison, including a + 27.9 FC in *Grem1* (gremlin 1; a bone morphogenetic protein (BMP) antagonist), *Sgcg* (gamma-sarcoglycan; + 10.9 FC), *Ildr2* (immunoglobulin-like domain containing receptor 2; + 7.7 FC) and *Bdnf* (encoding brain-derived neurotrophic factor (BDNF), + 7.4 FC). Downregulated transcripts include *Cyp1a1* (a member of the cytochrome P450 superfamily of enzymes involved in lipid metabolism; − 12.9 FC) and *Nefl* (neurofilament light, a component of the axonal cytoskeleton; − 8.1 FC).

Despite the statistically similar increased weights of bladders of diabetic and sucrose-treated rats, fewer transcripts (366) were significantly altered in sucrose-treated rat bladders compared with controls: 128 were upregulated and 238 downregulated (*p* < 0.05; Fig. 3C). As with the diabetic rats, *Grem1* (+ 4.2 FC) and *Ildr2* (+ 3.2 FC) were among the most upregulated transcripts in sucrose-treated rats versus controls. *Cped1* (cadherin like and PC-esterase domain containing 1) and *Pxmp4* (peroxisomal membrane protein 4) were most downregulated as assessed by FC (Suppl. Table 3). Paired-comparison of sucrose-treated rats with diabetic rats, revealed 1113 transcripts (411 upregulated and 702 downregulated) to be differentially expressed (*p* < 0.05 Fig. 3E; Suppl. Table 4) highlighting striking molecular differences between these two groups.

GennVenn analysis was performed to determine how many of the differentially expressed transcripts were exclusively located in specific paired comparison (Suppl. Fig. 2): 1298 of the 1467 differentially expressed transcripts were exclusively altered in the diabetic versus control comparison; 197 of the 366 altered transcripts were only altered in the sucrose-treated versus control comparison; and 169 transcripts were altered in both comparisons. qRT-PCR was performed to validate array data using a selection of upregulated, downregulated and unchanged transcripts and showed good reproducibility of array results (Supp. Fig. 3A–C) as well as immunohistochemistry for two of the upregulated molecules (BDNF: Supp. Fig. 3D–I and neurotrimin: Supp. Fig. 3J–O).

A bioinformatics approach using IPA was utilised to identify pathways in which multiple transcripts were differentially expressed. This revealed 42 and 27 significantly altered pathways in bladders of diabetic or sucrose-treated rats respectively compared with controls, and 27 overrepresented pathways in the sucrose-treated versus diabetic comparison (top 15 pathways shown in Fig. 3B, D, F; for all pathways: Suppl. Figs. 4, 5, and 6). Seven over-represented pathways (including: *hepatic fibrosi*s, *agranulocyte*/*granulocyte adhesion and diapedesis* and *leukocyte extravasation signaling*) were common to both diabetic versus control and sucrose versus control comparisons, compatible with common dysregulated fibrotic and inflammatory processes (Fig. 3B, D, F; Supp. Figs. 4, 5, and 6). Notable were the overrepresented pathways specific to the diabetic versus control comparison including ‘*inhibition of matrix metalloproteases* (Suppl. Fig. 4C) and ‘*axonal guidance signalling*’ (Suppl. Fig. 4D) pathways (Fig. 3B), thus highlighting the potential for DM-specific induced bladder dysfunction involving neural signalling and ECM regulation.

**Figure 4:**
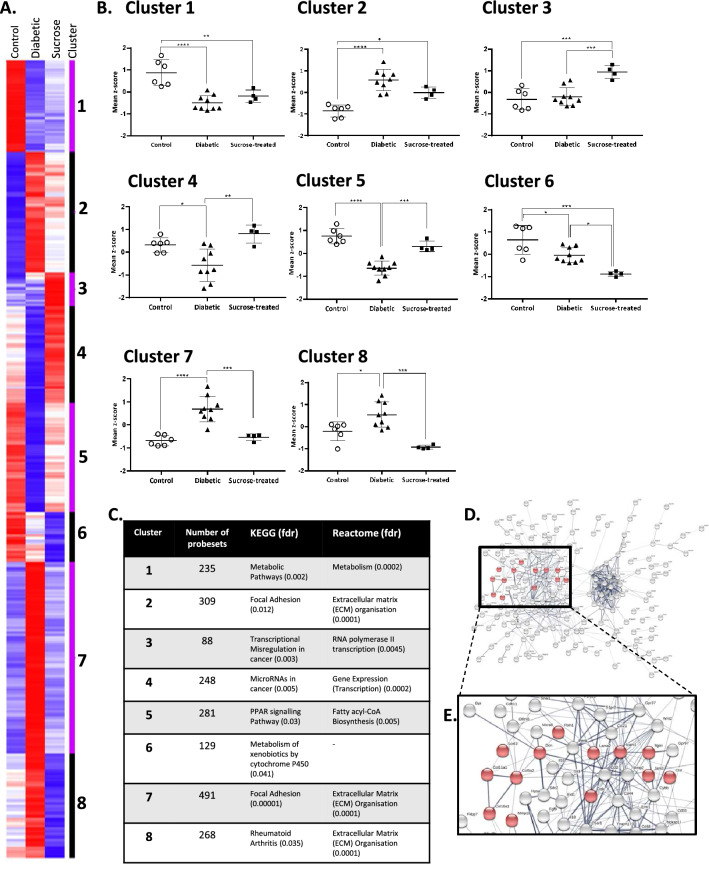
Cluster analysis demonstrates an altered pattern of transcript expression in the bladders of diabetic and sucrose-treated rats. κ-means clustering grouped transcripts into eight clusters (**A**) Rows are the mean transcript expression levels (Control *n* = 6; Diabetic *n* = 9; Sucrose-treated *n* = 4) denoted as the z‐score displayed in colourised (High (Red) to Low (Blue) expression) scale. Columns are experimental groups. See Supp. Fig. 7 for full cluster analysis including transcript names. (**B**) Analysis of the z-score profiles in each cluster. Data are expressed as the mean Cluster z-scores for individual animals in each group (mean ± standard deviation; one-way ANOVA followed by Tukey’s post-hoc test; *p < 0.05; **p < 0.01; ***p < 0.001; ****p < 0.0001). (**C**) The most significant over‐represented KEGG pathway and reactome are shown for each cluster (see Suppl. Fig. 8 for larger image. (**D**, **E**) STRING network analysis of Cluster 8 highlights interaction networks and the molecules for the most significant reactome pathway (‘*extracellular matrix organisation*’) are highlighted as red coloured and annotated nodes.

Clustering analysis of the probe sets passing filters of *p* < 0.05 and ± 1.3FC in any of the three comparisons separated the gene list into eight clusters based on similarity of expression profile across the three experimental conditions (Fig. 4A, B for the full and annotated Cluster: Supp. Fig. 7). Transcripts in Clusters 1 and 2 were significantly, and similarly, dysregulated in diabetic and sucrose-treated versus controls. Of note were the clusters in which differential expression levels were evident specifically in bladders from diabetic and sucrose-treated rats (Fig. 4A, B). For Cluster 3, the smallest cluster containing 88 probesets (Fig. 4C) the mean z-score was upregulated only in sucrose-treated rats. In comparison, Cluster 4 and 5 mean z-scores were downregulated, and Clusters 7 and 8 mean z-scores were upregulated specifically in diabetic rats. Three of the largest Clusters (2, 7 and 8) were enriched with transcripts associated with ECM organisation (Fig. 4C). For example, Cluster 8 (Fig. 4C–E; Suppl. Fig. 8) contained ECM-related transcripts including *Col5a2*, *Col16a1*, *Coll11a1*, *Ctsk*, *Dcn*, *Fbln1*, *Hpse*, *Lama2*, *Mmp2*, *Mmp19* and *Sdc2*. Given this, and the IPA result regarding the DM-specific overrepresentation of the ‘*Inhibition of matrix metalloproteases*’ pathway (Fig. 3B), we performed further qRT-PCR on genes from this IPA pathway (Suppl. Fig. 4C) and selected genes from Cluster 8 (Supp. Fig. 9: *Mmp2*, *Mmp14*, *Mmp15*, *Mmp16*, *Mmp17*, *Mmp19*, *Mmp23*, *Timp1*, *Timp2*, *Timp3*, *Lrp1 and Ctsk*). There were significant increases in levels of *Timp2* (*p* = 0.039), *Timp3* (*p* = 0.002) *and Mmp17* (*p* = 0.0253), with near significant increases in *Mmp14* (*p* = 0.0523) and *Mmp19* (*p* = 0.0537) in bladders from diabetic but not sucrose-treated animals, highlighting DM-specific dysregulation of ECM organisation.

In addition, we examined 16-week old *db*/*db* mice to ascertain if the bladder transcriptomic profile was similar in an established model of type 2 DM. At this time-point, urine output, blood glucose and body weight were significantly greater in *db*/*db* mice versus* db*/*lean* mice (Suppl. Table 5). The direction of changes in 18 out of 25 selected genes altered in the bladders of STZ-diabetic rats versus controls were replicated in the bladders of *db*/*db* mice compared with *db*/*lean* animals (Table 2), and 11 of these 18 genes were significantly altered (increased: *Arhgap1*; *Aspn*, *Bdnf*, *Cntf*, *Itga8*, *Lyve1*, *Mmp14*, *Mrc1*, *Tgfb2* and *Vcan*, and decreased: *Cyp1a1*).

**Table 2:** Transcriptional changes in the *db*/*db* mouse urinary bladder.

Probe ID	Gene symbol	Gene name	Rat (microarray data)		Mouse (qRT-PCR data)	
			Fold change (diabetic vs control)	*p* value	Fold-change (*db*/*db* vs lean)	*p* value
**Upregulated**						
1368677_at	*Bdnf*	Brain-derived neurotrophic factor	7.4	3.92E−05	3.4	0.05
1381504_at	*Aspn*	Asporin	4.8	1.08E−06	9.8	0.004
1388142_at	*Vcan*	Versican	3.8	8.27E−05	5.2	0.005
1378225_at	*Mmp14*	Matrix metallopeptidase 14	1.6	0.0113	3.1	0.0387
1368658_at	*Cntf*	Ciliary neurotrophic factor	2.8	0.0003	3.7	0.0464
1387172_a_at	*Tgfb2*	Transforming growth factor, beta 2	1.6	0.0004	2.2	0.0524
1383398_at	*Itga8*	Integrin, alpha 8	3.4	2.82E−06	2.4	0.0479
1382192_at	*Lyve1*	Lymphatic vessel endothelial hyaluronan receptor 1	3.0	3.43E−06	4.7	0.0003
1392648_at	*Mrc1*	Mannose receptor, C type 1	2.2	0.0006	4.5	0.0324
1393113_at	*Arhgap1*	Rho GTPase activating protein 1	2.5	0.0006	1.9	0.05
1370747_at	*Fgf9*	Fibroblast growth factor 9	1.4	0.0055	1.3	NS
1376410_at	*Mmp17*	Matrix metalloproteinase-17-like /// matrix metallopeptidase 17	1.9	0.0003	1.8	NS
1369113_at	*Grem1*	Gremlin 1	27.9	1.69E−07	− 1.3	NS
1374353_x_at	*Acta2*//*Actc1*	Actin, alpha 2, smooth muscle, aorta /// actin, alpha, cardiac muscle 1	2.5	2.15E−05	2.2	NS
1367631_at	*Ctgf*	Connective tissue growth factor	2.8	0.0003	2.3	NS
**Downregulated**						
1393069_at	*Sfrp5*	Secreted frizzled-related protein 5	− 2.1	8.04E−05	3.1	0.0002
1386881_at	*Igfbp3*	Insulin-like growth factor binding protein 3	− 2.7	1.17E−05	3.2	0.0197
1370269_at	*Cyp1a1*	Cytochrome P450, family 1, subfamily a, polypeptide 1	− 12.9	0.0001	− 1.4	0.3298
1369572_at	*Mcpt1*	Mast cell protease 1	− 3.1	0.0293	49.8	0.0483
1367845_at	*Nefm*	Neurofilament, medium polypeptide	− 1.8	0.0016	2.1	0.0535
1393271_at	*Ihh*	Indian hedgehog	− 2.2	1.51E−06	3.6	0.086
1369968_at	*Ptn*	Pleiotrophin	− 1.8	6.94E−05	− 1.1	NS
1387208_at	*Ngb*	Neuroglobin	− 3.1	7.57E−08	− 1.5	NS
1368990_at	*Cyp1b1*	Cytochrome P450, family 1, subfamily b, polypeptide 1	− 3.0	0.0018	1.6	NS
1367571_a_at	*Igf2*	Insulin-like growth factor 2	− 1.6	0.0017	− 1.3	NS

A comparison of microarray results with qRT-PCR of selected transcripts in the urinary bladder of *db*/*db* mice compared to age-matched lean controls (16 weeks) reveals some similarities and differences between the two models.

Collectively, these data revealed that whilst increased fluid intake induced similar increases in bladder weight in diabetic and sucrose-treated rats, distinct changes occurred in the pattern of gene expression in the bladder, which may represent a consequence of the metabolic effects of DM rather than the compensatory response to the increased urine load. Furthermore, replication of certain changes in the *db*/*db* mice highlights translation of these findings to a model of Type 2 DM.

### Fibrillar collagen is reduced in DSM of diabetic but not sucrose-treated rats

**Figure 5:**
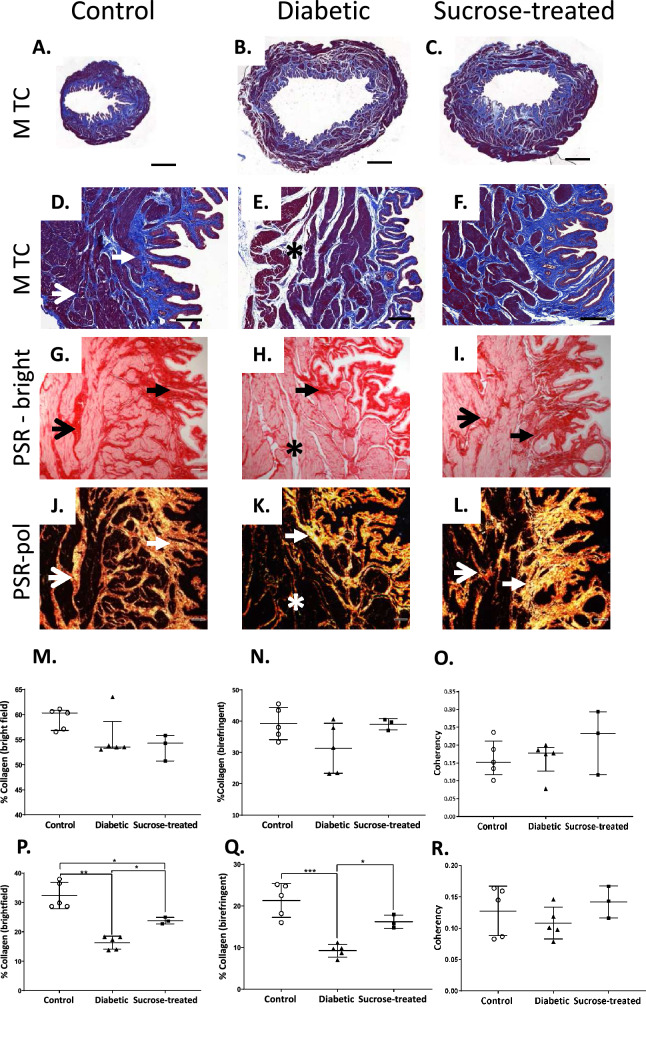
Fibrillar collagen is reduced in the detrusor muscle of diabetic but not sucrose-treated rats. Histological staining for collagen using Masson’s trichrome (MTC; **A**–**F**) and picrosirius red (PSR, **G**–**L**) revealed dense staining (blue, **A**–**F**; red (**G**–**I**) in the lamina propria (closed arrows) and surrounding muscle bundles (open arrows) in the control rat bladder. Expanded unstained regions around muscle bundles of diabetic rats were noted (**E**, **H** asterisks) these were not prominent in the sucrose-treated rats (**F**, **I**). Whilst PSR-stained collagen did not significantly change in the lamina propria (closed arrows; **G**–**I**, **M**, **N**), there was a notable loss in the detrusor muscle of diabetic rats (asterisks; **H**, **K**, **P**, **Q**) compared with controls (**G**, **J**) and sucrose-treated rats (**I**, **L**). (**J**–**L**) Fibrillar collagens appear as different colours when PSR-staining is viewed under polarized light, and were significantly reduced in the detrusor muscle of diabetic (**K**, **Q**) but not sucrose-treated (**L**, **Q**) rats. The organisation/alignment of birefringent collagen fibrils (‘coherency’) was not significantly different in any group (**O**, **R**). Data are expressed as (**M**, **O**) median ± interquartile range, analysed by Kruskal–Wallis followed by Dunn’s multiple comparison test or mean ± SD (**N**, **P**–**R**: control *n* = 5, diabetic *n* = 5; sucrose-treated *n* = 3**)** analysed by one way ANOVA followed by Tukey’s post hoc test (*p < 0.05; **p < 0.01; ***p < 0.001).

Our bioinfomatics analyses highlighted ECM organisation pathways, so we examined the localisation and organisation of total and fibrillar collagen (Fig. 5). Masson’s trichrome staining revealed a prominent collagenous network around DSM bundles and in the lamina propria of control (Fig. 5A, D) and sucrose-treated rats (Fig. 5C, F) but less collagen around DSM in diabetic rats (Fig. 5B, E asterisk). PSR-staining was examined under both bright field to visualise total collagen (Fig. 5G–I) and under polarised light, to assess birefringent organised fibrillar collagen (Fig. 5J–L). Whilst collagen staining did not change in the lamina propria (*p* > 0.05; Fig. 5M–O) expanded unstained regions between DSM bundles of diabetic rats were evident. There was a significant reduction in total (Fig. 5P) and birefringent (Fig. 5Q) collagen in DSM of diabetic rats versus untreated controls (asterisk; Fig. 5H, K, *p* < 0.01). Whilst there was some reduction in total collagen staining in DSM of sucrose-treated rats (Fig. 5I, P, *p* < 0.05) compared with controls, staining was significantly greater than in diabetic rats (Fig. 5P *p* < 0.05) and birefringent fibrillar collagen remained at control levels (Fig. 5L, Q, *p* > 0.05). Immunofluorescence analysis revealed collagen I expression was significantly reduced in diabetic DSM (Suppl. Fig. 10). The organisation/alignment of birefringent collagen fibrils (‘coherency’) in lamina propria or DSM was not significantly affected (Fig. 5L, O, *p* > 0.05).

### Altered biomechanical properties of the bladder in experimental DM

**Figure 6:**
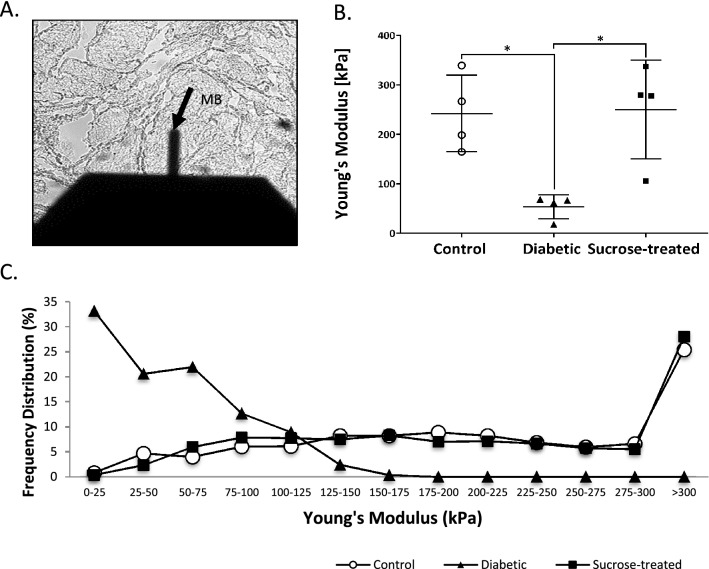
Detrusor muscle tissue from diabetic rats was significantly less stiff than tissue from control and sucrose-treated rats. (**A**) A representative bright-field image shows ex vivo tissue sections of detrusor muscle bundles investigated by atomic force microscopy (AFM) nanoindentation. (**B**) There was a significant reduction in tissue stiffness of detrusor muscle of diabetic rats compared with control and sucrose-treated rats, with a significant reduction in mean Young’s modulus (kPa; mean ± SD, analysed by one way ANOVA followed by Tukey’s post hoc test (control *n* = 4, diabetic *n* = 4; sucrose-treated *n* = 4; *p < 0.05) (**C**) Frequency distribution profile of Young’s modulus readings obtained from tissue of diabetic rats compared to the similar profiles of readings obtained from control and sucrose-treated rat bladders.

Given that fibrillar collagens play vital structural roles and provide tensile strength to the bladder^26^ we next investigated whether the nanomechanical properties of the detrusor muscle were altered using AFM, a powerful tool for characterising ex vivo tissue. The elastic properties of tissue in sections of DSM were investigated by nanoindentation using a 5 mm borosilicate sphere attached to a calibrated cantilever (Fig. 6A). Deflections of the cantilever were used to generate Young’s modulus values, these being a measure of the localised resistance to deformation when a specific force is applied. There was a significant reduction in the stiffness of diabetic DSM versus controls (Fig. 6B: Control: 242.2 ± 77.3 kPa; diabetic 53.5 ± 24.2 kPa; *p* < 0.05). Interestingly, this change was not evident in bladders from sucrose-treated rats (250.2 ± 100 kPa *p* > 0.05), which were not significantly different to controls. The marked difference in tissue rigidity can be seen in the frequency distribution profile plot of Young’s modulus readings from bladder tissue of diabetic rats compared with the almost identical profiles obtained from tissues from control and sucrose-treated rats, where 0.8% and 0.4% of measurements were < 25 kPa, and 25.4% and 28.1% were > 300 kPa in the control and sucrose-treated rats respectively (Fig. 6C). This contrasts with the bladder samples from diabetic rats in which 33.2% of the readings were < 25 kPa, and no readings were > 175 kPa (Fig. 6C). This highlights that detrusor muscle tissue from diabetic rats was significantly less stiff than control and sucrose-treated rats. We suggest that remodelling of the bladder architecture and DM-associated decrease in collagen fibres may contribute towards this decrease in tissue stiffness.

## Discussion

Our results support the hypothesis that the dysregulated transcriptome in diabetic bladders in an experimental model of long-term DM represents a summation of that induced by polyuria with that induced by the diabetic milieu. This duration of DM is associated with functional changes such as decreases in peak voiding pressure and inadequate voiding^9^, consistent with features of clinical diabetic cystopathy. In diabetic rat bladders, bioinformatics highlighted aberrations of ECM regulators, and there was reduced birefringent fibrillar collagen in DSM of diabetic but not sucrose-treated rats. Strikingly, AFM detected reduced tissue stiffness in diabetic, but not sucrose-treated, bladders. We postulate these changes contribute to dysfunctional voiding in DM. Diabetic and sucrose-treated rats had similar DSM hypertrophy and increased bladder weights versus control organs. The impact on bladder transcriptomes was however not identical, and by comparing differences between the groups we hoped to gain insight into the pathogenesis of diabetic cystopathy and distinguish a characteristic molecular signature for DM-specific changes.

Some of the altered transcripts and over-represented canonical pathways were similarly dysregulated in diabetic and sucrose-treated groups compared with controls, and thus most likely represent the adaptive response to bladder distension associated with polyuria. DM-specific changes included transcripts associated with axonal signalling and ECM regulation pathways. Thus, these pathways may be differentially regulated by the direct (e.g. metabolic and/or vascular consequences of hyperglycaemia/hypoinsulinaemia) or indirect (e.g. through glycative and oxidative stress) consequences of DM, rather than the physical adaption to polyuria.

One of the most important findings of our study is that DM led to alterations in the bladder ECM, a dynamic structure, remodelled through coordinated transcription, translation, post-translational degradation and modifications^26,27^. Glycosaminoglycans are abundant in the urothelium, constituting a protective barrier to protect against urine toxicity and infections; elastin enables recoil of the bladder after micturition; whilst rigidity and compliance is conferred largely by collagen I and III^28^. Altered mRNA and protein levels of collagen in diabetic rodent bladders (1 and 8 weeks post-STZ^17–19^ have been found using various techniques including histological staining with Masson’s trichrome and immunolabelling techniques^11,13,28^. We also observed a decrease in collagen, particularly within the DSM layer, using Masson’s trichrome and immunocytochemistry and extended these observations by assessing PSR-staining under polarised light to examine birefringent organised collagen, and found this markedly reduced in diabetic bladders. This contrasts with observations in diabetic kidneys from Wistar rats administered STZ for 6–8 weeks which display enhanced fibrosis and collagen deposition by Periodic Acid Schiff and Masson’s trichrome staining respectively^29,30^.

Large collagen I fibrils uncoil during the bladder filling phase to allow the accommodation of urine with little change in intravesical pressure^31^, thus changes in fibrillar collagen content will cause functional changes in tensile strength, resistance to distension and compliance of the bladder. A decrease in collagen around DSM bundles may affect the overall contractility of the detrusor because collagen fibrils play an important role in intercellular transmission of force. Tissue stiffness plays a crucial role in cell phenotype and cell behaviour can change on rigid, or soft, ECM, e.g. increased ECM stiffness increases invasiveness of cancer cells^32^. Using localised AFM nanoindentation we found a significant difference in the stiffness of DSM of diabetic, but not the sucrose-treated, rats versus controls.

Reduced ECM in Ehlers-Danlos syndrome (which is associated with mutations impacting on fibrillar collagens and fragility of connective tissue^33^ and in Marfan syndrome (associated with mutations in fibrillin-1) have been linked with an increased risk of urinary tract complications including vesico-ureteric reflux, diverticula^34^ and rupture from overdistension^35^. Whilst conversely, increased fibrosis and collagen deposition during bladder outflow obstruction^36,37^ has been associated with decreased compliance and reduced capacity to store urine.

Alterations in levels or activity of matrix metalloproteinases (MMPs) and tissue inhibitor of metalloproteinases (TIMPs) can lead to excessive production of, or increased proteolysis of ECM components, and tight regulation of ECM is thus critical to bladder integrity and function. ECM molecules modulate cell signalling, tension and phenotype through interactions with integrins and other cell adhesion receptors, and also acts as a reservoir of sequestered growth factors (e.g. VEGF, BMP4, TGF-β1) which are released by MMPs^38,39^. Initially it does seem counterintuitive to observe decreased collagen expression in association with overrepresentation of the ‘*Inhibition of matrix metalloproteases*’ IPA pathway and increased *Timp2 and Timp3* mRNA as the classical role for TIMPs would suggest that upregulated TIMPs inhibit MMPs, and reduce ECM proteolysis leading to ECM accumulation/fibrosis. This could suggest that at the 16 week timepoint we are measuring a late compensatory change in TIMP levels in response to an earlier increase in MMP levels or activity. Alternatively, it may reflect the complex dichotomous role that TIMPS and MMPs play in ECM turnover in different tissues and conditions. For example, whilst *Timp3*^−/−^ mice have increased MMP activity, and reduced collagen levels in joints^40^and lungs^41^, they develop an age-related chronic tubulointerstitial fibrosis, with increased levels of type I collagen in the kidney (despite a greater activation of MMP2^42^ and increased fibrosis in the heart^43^. The ‘*Inhibition of matrix metalloproteases*’ IPA pathway (Supp. Fig. 4C) also includes transmembrane MT-MMPs (MMP-14, -15, -16, and -24) and the GPI-lined MMP-17. Most are able to activate proMMP-2 and MT1-MMP (MMP-14) which has direct proteolytic activity on collagen^44^. While transcriptional changes and loss of collagen highlight significant disruption of ECM homeostasis (Suppl. Fig. 11), future studies should elucidate the temporal changes in ECM, and ECM regulatory molecules at the gene, protein and activity level in the bladder in DM, as well as functional cystometry to identify therapeutic targets and intervention points. We hypothesise that loss of fibrillar collagen through dysregulated ECM regulatory pathways in DM, and increased flaccidity of the bladder wall contributes to increased compliance and/or inadequate emptying of the bladder observed in diabetic cystopathy.

An alternative hypothesis to the DM milieu being directly harmful to bladder cells is that DM-induced arteriosclerosis causes bladder ischaemia and subsequent bladder cell damage. It has been reported that experimental models of atherosclerosis in rabbits and rats are associated with bladder ischaemia and altered mechanical properties^45,46^ that cardiovascular risk factors in human populations correlate with urinary tract symptoms and bladder storage capacity^47,48^ These studies, however, do not primarily address DM per se. Moreover, as recently reviewed^49^, rats are resistant to acquiring atherosclerosis unless they have undergone, as examples, physical vascular damage and/or exposure to special diets to increase blood lipids. Neither manipulation was undertaken in the current study, and lipids (cholesterol and triglycerides) were not significantly increased in our rat DM model (Table 1). Thus, while we cannot formally exclude atherosclerosis of bladder arteries as having a pathogenic role in our model, this appears to be a less likely hypothesis than invoking the direct metabolic effects of DM on bladder tissues.

The ‘a*xonal guidance signaling*’ canonical pathway was also overrepresented exclusively in the diabetic versus control comparison (Fig. 3B); this pathway includes transcripts for growth factors and guidance cues such as *Bdnf*, *EphA4*, *Ngf* and *Slit3* (Suppl. Fig. 4A, D). The micturition cycle is tightly controlled by neural systems that interact locally, spinally and supraspinally^50^. Urinary continence therefore depends on highly coordinated activity between the peripheral and central nervous system, and the passive and active contractile properties of the bladder wall in detrusor muscle cycling^50,51^. Structural and functional neurological changes have been implicated in the pathogenesis of diabetic bladder dysfunction^52^. One of the most down-regulated transcripts in the bladder of diabetic, but not sucrose-treated, rats was *Nefl*, which encodes neurofilament light, an integral component of the axonal cytoskeleton Neurofilaments govern axonal calibre and thus impact on nerve conduction velocity. Loss of neurofilament has previously been reported in experimental diabetic neuropathy in peripheral nervous system of STZ-diabetic rats^53^.

Reduced production of neurotrophic factors by target end-organs leads to reduced retrograde axonal transport of those growth factors and contributes to the pathogenesis of diabetic neuropathy and local effects^54^. For example, a decrease in NGF levels in the bladder and associated dorsal root ganglia (DRG) has been correlated with the progression of diabetic cystopathy^55^. A gene therapy approach in which a HSV-1 vector expressing the *Ngf* gene was injected into bladder walls of diabetic rats, ameliorated the diabetes-associated deficits in NGF levels in both the bladder and lumbosacral DRG, and improved the hypoactive bladder phenotype compared with diabetic rats injected with a control-vector^56^. In our study, *Bdnf* upregulation was detected in bladders of both STZ-diabetic rats and *db*/*db* mice. Increased urinary BDNF levels are increased in many other conditions associated with urinary tract symptoms including interstitial cystitis^57^, overactive bladder^58^, inflammation^59^ and spinal cord injury^60^. Local transgenic overexpression of BDNF in otherwise healthy rat bladders induced choline acetyltransferase expression at cholinergic nerve terminals, and increased bladder activity with higher voiding pressure and reduced reflex voiding intervals^61^. It will be interesting to elucidate the functional impact of increased BDNF in diabetic cystopathy in future mechanistic studies.

The results presented in the current paper serve as an informative stepping stone towards understanding the complex pathobiology of diabetic cystopathy. A caveat of all transcriptomic analyses is that protein expression may not exactly parallel the spatial and/or temporal changes in the RNA levels. Accordingly, in future, an even more powerful experimental strategy will be to undertake single cell RNA sequencing studies^62^, to correlate levels of individual transcripts with unique tissue compartments, in combination with proteomic analysis^20,63^ to quantify and locate the many up- or down-regulated molecules in models of diabetic cystopathy.

## Summary

Our study provides insight into the pathogenesis of bladder remodelling in both polyuric and diabetic cystopathies. The results in particular point to an altered ECM and reduced tissue rigidity in DM, being a central cause of the incomplete voiding reported in people who have bladder damage in long term DM. Future mechanistic experiments, for example local targeting of MMP activity, are needed to prove whether modulation of collagen and reduction in tissue stiffness contributes to the transition from a compensated to decompensated atonic bladder.

## Supplementary Information

Supplementary Information 1.

Supplementary Figure 7.

## Data Availability

The datasets generated are deposited in ArrayExpress (E-MTAB-9255).

## References

[1] Gnudi L, Coward RJM, Long DA (2016). Diabetic nephropathy: Perspective on novel molecular mechanisms. Trends Endocrinol. Metab..

[2] Frimodt-Moller C (1978). Diabetic cystopathy. A review of the urodynamic and clinical features of neurogenic bladder dysfunction in diabetes mellitus. Dan Med. Bull..

[3] Karoli R, Bhat S, Fatima J, Priya S (2014). A study of bladder dysfunction in women with type 2 diabetes mellitus. Indian J. Endocrinol. Metab..

[4] Frimodt-Moller C (1980). Diabetic cystopathy: Epidemiology and related disorders. Ann. Intern. Med..

[5] Powell CR (2014). Is the diabetic bladder a neurogenic bladder? Evidence from the literature. Curr. Bladder Dysfunct. Rep..

[6] Golbidi S, Laher I (2010). Bladder dysfunction in diabetes mellitus. Front. Pharmacol..

[7] Liu RT, Chung MS, Lee WC, Chang SW, Huang ST, Yang KD, Chancellor MB, Chuang YC (2011). Prevalence of overactive bladder and associated risk factors in 1359 patients with type 2 diabetes. Urology.

[8] Kaplan SA, Te AE, Blaivas JG (1995). Urodynamic findings in patients with diabetic cystopathy. J. Urol..

[9] Daneshgari F, Huang X, Liu G, Bena J, Saffore L, Powell CT (2006). Temporal differences in bladder dysfunction caused by diabetes, diuresis, and treated diabetes in mice. Am. J. Physiol. Regul. Integr. Comp. Physiol..

[10] Xiao N, Huang Y, Kavran M, Elrashidy RA, Liu G (2015). Short-term diabetes- and diuresis-induced alterations of the bladder are mostly reversible in rats. Int. J. Urol..

[11] Eika B, Levin RM, Longhurst PA (1994). Comparison of urinary bladder function in rats with hereditary diabetes insipidus, streptozotocin-induced diabetes mellitus, and nondiabetic osmotic diuresis. J. Urol..

[12] Daneshgari F, Liu G, Birder L, Hanna-Mitchell AT, Chacko S (2009). Diabetic bladder dysfunction: Current translational knowledge. J. Urol..

[13] Liu G, Daneshgari F (2006). Temporal diabetes- and diuresis-induced remodeling of the urinary bladder in the rat. Am. J. Physiol. Regul. Integr. Comp. Physiol..

[14] Liu G, Li M, Vasanji A, Daneshgari F (2011). Temporal diabetes and diuresis-induced alteration of nerves and vasculature of the urinary bladder in the rat. BJU Int..

[15] Daneshgari F, Liu G, Imrey PB (2006). Time dependent changes in diabetic cystopathy in rats include compensated and decompensated bladder function. J. Urol..

[16] Wang Y, Deng GG, Davies KP (2016). Novel insights into development of diabetic bladder disorder provided by metabolomic analysis of the rat nondiabetic and diabetic detrusor and urothelial layer. Am. J. Physiol. Endocrinol. Metab..

[17] Hipp JD, Davies KP, Tar M, Valcic M, Knoll A, Melman A, Christ GJ (2007). Using gene chips to identify organ-specific, smooth muscle responses to experimental diabetes: Potential applications to urological diseases. BJU Int..

[18] Kanika ND, Chang J, Tong Y, Tiplitsky S, Lin J, Yohannes E, Tar M, Chance M, Christ GJ, Melman A (2011). Oxidative stress status accompanying diabetic bladder cystopathy results in the activation of protein degradation pathways. BJU Int..

[19] Yohannes E, Chang J, Christ GJ, Davies KP, Chance MR (2008). Proteomics analysis identifies molecular targets related to diabetes mellitus-associated bladder dysfunction. Mol. Cell. Proteom.: MCP.

[20] Kassab S, Begley P, Church SJ, Rotariu SM, Chevalier-Riffard C, Dowsey AW, Phillips AM, Zeef LAH, Grayson B, Neill JC (2019). Cognitive dysfunction in diabetic rats is prevented by pyridoxamine treatment. A multidisciplinary investigation. Mol. Metab..

[21] Rezakhaniha R, Agianniotis A, Schrauwen JT, Griffa A, Sage D, Bouten CV, van de Vosse FN, Unser M, Stergiopulos N (2012). Experimental investigation of collagen waviness and orientation in the arterial adventitia using confocal laser scanning microscopy. Biomech. Model. Mechanobiol..

[22] Li C, Wong WH (2001). Model-based analysis of oligonucleotide arrays: Expression index computation and outlier detection. Proc. Natl. Acad. Sci. U. S. A..

[23] Bolstad BM, Irizarry RA, Astrand M, Speed TP (2003). A comparison of normalization methods for high density oligonucleotide array data based on variance and bias. Bioinformatics.

[24] Storey JD, Tibshirani R (2003). Statistical significance for genomewide studies. Proc. Natl. Acad. Sci. U. S. A..

[25] Long DA, Kolatsi-Joannou M, Price KL, Dessapt-Baradez C, Huang JL, Papakrivopoulou E, Hubank M, Korstanje R, Gnudi L, Woolf AS (2013). Albuminuria is associated with too few glomeruli and too much testosterone. Kidney Int..

[26] Aitken KJ, Bagli DJ (2009). The bladder extracellular matrix. Part I: Architecture, development and disease. Nat. Rev. Urol..

[27] Zeltz C, Gullberg D (2014). Post-translational modifications of integrin ligands as pathogenic mechanisms in disease. Matrix Biol.: J. Int. Soc. Matrix Biol..

[28] Eika B, Levin RM, Longhurst PA (1992). Collagen and bladder function in streptozotocin-diabetic rats: Effects of insulin and aminoguanidine. J. Urol..

[29] Hodrea J, Balogh DB, Hosszu A, Lenart L, Besztercei B, Koszegi S, Sparding N, Genovese F, Wagner LJ, Szabo AJ (2020). Reduced O-GlcNAcylation and tubular hypoxia contribute to the antifibrotic effect of SGLT2 inhibitor dapagliflozin in the diabetic kidney. Am. J. Physiol. Ren. Physiol..

[30] Ren XJ, Guan GJ, Liu G, Zhang T, Liu GH (2009). Effect of activin A on tubulointerstitial fibrosis in diabetic nephropathy. Nephrology (Carlton).

[31] Cheng F, Birder LA, Kullmann FA, Hornsby J, Watton PN, Watkins S, Thompson M, Robertson AM (2018). Layer-dependent role of collagen recruitment during loading of the rat bladder wall. Biomech. Model. Mechanobiol..

[32] Kirschmann DA, Seftor EA, Fong SF, Nieva DR, Sullivan CM, Edwards EM, Sommer P, Csiszar K, Hendrix MJ (2002). A molecular role for lysyl oxidase in breast cancer invasion. Can. Res..

[33] Beighton P, De Paepe A, Steinmann B, Tsipouras P, Wenstrup RJ (1998). Ehlers-Danlos syndromes: Revised nosology, Villefranche, 1997 Ehlers-Danlos National Foundation (USA) and Ehlers-Danlos support group (UK). Am. J. Med. Genet..

[34] Clunie GJ, Mason JM (1962). Visceral diverticula and the Marfan syndrome. Br. J. Surg..

[35] Jorion JL, Michel M (1999). Spontaneous rupture of bladder diverticula in a girl with Ehlers-Danlos syndrome. J. Pediatr. Surg..

[36] Inaba M, Ukimura O, Yaoi T, Kawauchi A, Fushiki S, Miki T (2007). Upregulation of heme oxygenase and collagen type III in the rat bladder after partial bladder outlet obstruction. Urol. Int..

[37] Qiao LY, Xia C, Shen S, Lee SH, Ratz PH, Fraser MO, Miner A, Speich JE, Lysiak JJ, Steers WD (2018). Urinary bladder organ hypertrophy is partially regulated by Akt1-mediated protein synthesis pathway. Life Sci..

[38] Hynes RO (2009). The extracellular matrix: Not just pretty fibrils. Science.

[39] Hynes RO, Naba A (2012). Overview of the matrisome—An inventory of extracellular matrix constituents and functions. Cold Spring Harb. Perspect. Biol..

[40] Sahebjam S, Khokha R, Mort JS (2007). Increased collagen and aggrecan degradation with age in the joints of Timp3(-/-) mice. Arthr. Rheum..

[41] Leco KJ, Waterhouse P, Sanchez OH, Gowing KL, Poole AR, Wakeham A, Mak TW, Khokha R (2001). Spontaneous air space enlargement in the lungs of mice lacking tissue inhibitor of metalloproteinases-3 (TIMP-3). J. Clin. Invest..

[42] Kassiri Z, Oudit GY, Kandalam V, Awad A, Wang X, Ziou X, Maeda N, Herzenberg AM, Scholey JW (2009). Loss of TIMP3 enhances interstitial nephritis and fibrosis. J. Am. Soc. Nephrol..

[43] Kassiri Z, Defamie V, Hariri M, Oudit GY, Anthwal S, Dawood F, Liu P, Khokha R (2009). Simultaneous transforming growth factor beta-tumor necrosis factor activation and cross-talk cause aberrant remodeling response and myocardial fibrosis in Timp3-deficient heart. J. Biol. Chem..

[44] Li Z, Takino T, Endo Y, Sato H (2017). Activation of MMP-9 by membrane type-1 MMP/MMP-2 axis stimulates tumor metastasis. Cancer Sci..

[45] Azadzoi KM, Tarcan T, Siroky MB, Krane RJ (1999). Atherosclerosis-induced chronic ischemia causes bladder fibrosis and non-compliance in the rabbit. J. Urol..

[46] Nomiya M, Yamaguchi O, Akaihata H, Hata J, Sawada N, Kojima Y, Andersson KE (2014). Progressive vascular damage may lead to bladder underactivity in rats. J. Urol..

[47] Lee B, Lee SW, Kang HR, Kim DI, Sun HY, Kim JH (2018). Relationship between lower urinary tract symptoms and cardiovascular risk scores including Framingham risk score and ACC/AHA risk score. Neurourol. Urodyn..

[48] Monaghan TF, Miller CD, Agudelo CW, Rahman SN, Everaert K, Birder LA, Wein AJ, Weiss JP, Lazar JM (2021). Cardiovascular risk independently predicts small functional bladder storage capacity. Int. Urol. Nephrol..

[49] Zhao Y, Qu H, Wang Y, Xiao W, Zhang Y, Shi D (2020). Small rodent models of atherosclerosis. Biomed. Pharmacother..

[50] de Groat WC, Griffiths D, Yoshimura N (2015). Neural control of the lower urinary tract. Compr. Physiol..

[51] Young JS, Johnston L, Soubrane C, McCloskey KD, McMurray G, Eccles R, Fry CH (2013). The passive and active contractile properties of the neurogenic, underactive bladder. BJU Int..

[52] Vinik AI, Maser RE, Mitchell BD, Freeman R (2003). Diabetic autonomic neuropathy. Diabetes Care.

[53] Yagihashi S, Kamijo M, Watanabe K (1990). Reduced myelinated fiber size correlates with loss of axonal neurofilaments in peripheral nerve of chronically streptozotocin diabetic rats. Am. J. Pathol..

[54] Fernyhough P, Diemel LT, Brewster WJ, Tomlinson DR (1995). Altered neurotrophin mRNA levels in peripheral nerve and skeletal muscle of experimentally diabetic rats. J. Neurochem..

[55] Sasaki K, Chancellor MB, Phelan MW, Yokoyama T, Fraser MO, Seki S, Kubo K, Kumon H, Groat WC, Yoshimura N (2002). Diabetic cystopathy correlates with a long-term decrease in nerve growth factor levels in the bladder and lumbosacral dorsal root Ganglia. J. Urol..

[56] Sasaki K, Chancellor MB, Goins WF, Phelan MW, Glorioso JC, de Groat WC, Yoshimura N (2004). Gene therapy using replication-defective herpes simplex virus vectors expressing nerve growth factor in a rat model of diabetic cystopathy. Diabetes.

[57] Jiang YH, Liu HT, Kuo HC (2014). Decrease of urinary nerve growth factor but not brain-derived neurotrophic factor in patients with interstitial cystitis/bladder pain syndrome treated with hyaluronic acid. PLoS ONE.

[58] Wang LW, Han XM, Chen CH, Ma Y, Hai B (2014). Urinary brain-derived neurotrophic factor: A potential biomarker for objective diagnosis of overactive bladder. Int. Urol. Nephrol..

[59] Oddiah D, Anand P, McMahon SB, Rattray M (1998). Rapid increase of NGF, BDNF and NT-3 mRNAs in inflamed bladder. NeuroReport.

[60] Vizzard MA (2000). Changes in urinary bladder neurotrophic factor mRNA and NGF protein following urinary bladder dysfunction. Exp. Neurol..

[61] Kashyap MP, Pore SK, de Groat WC, Chermansky CJ, Yoshimura N, Tyagi P (2018). BDNF overexpression in the bladder induces neuronal changes to mediate bladder overactivity. Am. J. Physiol. Ren. Physiol..

[62] Yu Z, Liao J, Chen Y, Zou C, Zhang H, Cheng J, Liu D, Li T, Zhang Q, Li J (2019). Single-cell transcriptomic map of the human and mouse bladders. J. Am. Soc. Nephrol..

[63] Park EC, Lim JS, Kim SI, Lee SY, Tak YK, Choi CW, Yun S, Park J, Lee M, Chung HK (2018). Proteomic analysis of urothelium of rats with detrusor overactivity induced by bladder outlet obstruction. Mol. Cell. Proteom. MCP.

