# Supplementary material for: Experimental long-term diabetes mellitus alters the transcriptome and biomechanical properties of the rat urinary bladder
Source: Sci Rep. 2021 Jul 30;11:15529. doi: 10.1038/s41598-021-94532-7 (PMC8324824; doi:10.1038/s41598-021-94532-7)
Supplement: Supplementary file 1 — Supplementary Information 1. [file 41598_2021_94532_MOESM1_ESM.pdf]

Supplementary Information: Supplementary Tables, Figures and Legends

Experimental long-term diabetes mellitus alters the transcriptome and biomechanical properties of the rat urinary bladder

Emad A. Hindi[1,2], Craig J. Williams[3], Leo A. H. Zeef[1], Filipa M. Lopes[1], Katie Newman[1], Martha M.M. Davey[1], Nigel W. Hodson[1], Emma N. Hilton[1], Jennifer L. Huang[4], Karen L. Price[4], Neil A. Roberts[1], David A. Long[4], Adrian S. Woolf[1,5] & Natalie J. Gardiner[1*]

Supplementary Table 1. Primer details for qRT-PCR

Rat			
**Gene**	**Taqman Primer**		
Grem1	Rn01509832_m1		
Nefl	Rn00582365_m1		
Nefm	Rn00566763_m1		
Nefh	Rn00709325_m1		
Prph	Rn00688569_g1		
TuubIII	Rn00594933_m1		
Ngb	Rn00583724_m1		
Bdnf	Rn02531967_s1		
Ntrk2	Rn01441749_m1		
Ngfr	Rn00561634m1		
Ptn	Rn00567035_m1		
Ntm	Rn01525004_m1		
Actb	Rn00667869_m1		
Ppib	Rn03302274_m1		
Lrp1	Rn01503901_m1		
Mmp2	Rn01538168_m1		
Mmp14	Rn00579172_m1		
Mmp15	Rn01536925_m1		
Mmp16	Rn00490660_m1		
Mmp17	Rn01499864_m1		
Mmp19	Rn01756324_m1		
Mmp23	Rn00585994_m1		
Timp1	Rn01430875_g1		
Timp2	Rn01460081_m1		
Timp3	Rn00441826_m1		
Ctsk	Rn00580723_m1		
**Mouse**			
**Gene**	**Forward Primer**	**Reverse Primer**	**Amplicon Size**
Acta2	GCT ATT CAG GCT GTG CTG TC	GGT AGT CGG TGA GAT CTC GG	163bp
Arhgap1	TTG TGG AAG TAG CCG GTG AT	CTT GTT GTC ACT GGT CAG GC	188bp
Aspn	ACA ACG GGA TAG AAC CAG GG	GTT GTT TCC AAG ACC CAG CC	200bp
Bdnf	TGG CTG ACA CTT TTG AGC AC	GTT TGC GGC ATC CAG GTA AT	188bp
Cntf	CCA CAG GCA CAA AAT CCA CA	TCC CAG GAA ACA AGT GAG CT	176bp
Ctgf	TGC CAG TGG AGT TCA AAT GC	GTG TCC CTT ACT TCC TGG CT	169bp
Cyp1a1	CCA TGA TGA CCA AGA GCT GC	TGG CCC TTC TCA AAT GTC CT	207bp
Cyp1b1	GAC GAT GCG GAG TTC CTA GA	AAG TTG CTG AAG TTG CGG TT	164bp
Fgf9	GAC AGT GGA CTC TAC CTC GG	CCG TTT AGT CCT GGT CCC TT	210bp
Grem1	TAG AGG CCA GAA GAA CCA GC	CAA AGC CAA CTA CAG CCC TG	216bp
Igf2	GGG ACG TGT CTA CCT CTC AG	ATG ACG TTT GGC CTC TCT GA	191bp
Igfbp3	CGT CCA CAT CCC AAA CTG TG	TGA GGC AAT GTA CGT CGT CT	158bp
Ihh	AGG ACC GTC TGA ACT CAC TG	CAC GGT CTG AGG TGG TGA TA	153bp
Itga8	AGA AAT GAG GGA GAA GGG GC	CGA GGA ACA GCA AAT CGG AG	215bp
Lyve1	ACT TGC AGC TAT GGA TGG GT	GAA AAC TCT GTT GCG GGT GT	233bp
Mcpt1	TAA TTC CCT TGC CTG GTC CC	GGA ACT TCC CAC ACA GAC CT	194bp
Mmp14	GTG ACG GGA ACT TTG ACA CC	TTT GCC ATC CTT CCT CTC GT	179bp
Mmp17	AAG GCA CCT ACC CAG AAG TC	GAG ACC CAC AAT GCT CTC CT	233bp
Mrc1	TGG ATG GAT GGG AGC AAA GT	GCT GCT GTT ATG TCT CTG GC	165bp
Nefm	CTA TGC CCA AAT CAC CCG TG	CTT CTC TTT CAC CGG GGA CT	206bp
Ngb	CGC CCG GAG TCA GAG CTG AT	GTT GGT CAC TGC AGC ATC AA	228bp
Ptn	GTC CCA GCA ATA TCA GCA GC	CAC ACA CTC CAC TGC CAT TC	159bp
Sfrp5	CAC TCA GAC ACC CAG GTC TT	AGA TCT TGG TCA CTG GAG GC	241bp
Tgfb2	AAG CAA GCC GGT GAA ATG TT	TCA TGC TGG CTT CTA GAC CC	155bp
Vcan	CCG AAG CAG AGT GTA CAA GC	ATC AGG GAG AGG GAA GCA TG	218bp

Supplementary Table 2: The ten most altered transcripts (by fold change) in the urinary bladder of diabetic rats compared to control rats. Transcripts (with known gene symbols) were sorted according to fold-change (p<0.0)

Diabetic vs Control											
**Upregulated**						**Down regulated**					
**Probeset**	**Gene Symbol**	**Gene Title**	**Fold-change**	**fdr**	**p value**	**Probeset**	**Gene Symbol**	**Gene Title**	**Fold-change**	**fdr**	**p value**
1369113_at	***Grem1***	gremlin 1	**27.9**	0.000524	1.69E-07	1370269_at	***Cyp1a1***	cytochrome P450, family 1, subfamily a, polypeptide 1	**-12.9**	0.014215	0.000131
1374683_at	***Sgcg***	sarcoglycan, gamma (dystrophin-associated glycoprotein)	**10.9**	1.35E-05	8.70E-10	1370059_at	***Nefl***	neurofilament, light polypeptide	**-8.1**	0.000218	2.81E-08
1384035_at	***Ildr2***	immunoglobulin-like domain containing receptor 2	**7.7**	0.00048	1.39E-07	1387319_at	***Ccl11***	chemokine (C-C motif) ligand 11	**-6.3**	0.00101	6.29E-07
1368677_at	***Bdnf***	brain-derived neurotrophic factor	**7.4**	0.007265	3.92E-05	1370355_at	***Scd1***	stearoyl-Coenzyme A desaturase 1	**-4.4**	0.002957	7.23E-06
1374166_at	***Adprhl1***	ADP-ribosylhydrolase like 1	**4.9**	0.003929	1.06E-05	1371260_at	***Mcpt2***	mast cell protease 2	**-3.7**	0.213053	0.019379
1381504_at	***Aspn***	asporin	**4.8**	0.001475	1.08E-06	1369572_at	***Mcpt1***	mast cell protease 1	**-3.1**	0.260783	0.029299
1384564_at	***Fam159b***	family with sequence similarity 159, member B	**4.4**	0.001733	1.70E-06	1387208_at	***Ngb***	neuroglobin	**-3.1**	0.000392	7.57E-08
1379022_at	***Adamts8***	ADAM metallopeptidase with thrombospondin type 1 motif, 8	**4.3**	0.019393	0.000222	1370363_at	***Ces1d***	carboxylesterase 1D	**-3.0**	0.027377	0.000411
1390051_at	***Mtus2***	microtubule associated tumor suppressor candidate 2	**3.9**	0.002149	3.70E-06	1368990_at	***Cyp1b1***	cytochrome P450, family 1, subfamily b, polypeptide 1	**-3.0**	0.060406	0.001777
1388142_at	***Vcan***	versican	**3.8**	0.011128	8.27E-05	1384023_at	***LOC100909485 /// Spin2a***	spindlin-2-like /// spindlin family, member 2A	**-2.9**	0.004483	1.66E-05

Supplementary Table 3: The ten most altered transcripts (by fold change) in the urinary bladder of sucrose-treated rats compared to control rats
Transcripts (with known genes) were sorted according to fold-change ($p<0.05$)

Sucrose-treated vs Control												
**Upregulated**							**Down regulated**					
**Probeset**	**Gene Symbol**	**Gene Title**	**Fold-change**	**fdr**	**p value**		**Probeset**	**Gene Symbol**	**Gene Title**	**Fold-change**	**fdr**	**p value**
1369113_at	***Grem1***	gremlin 1	**4.2**	0.93595	0.011098		1392074_at	***Cped1***	cadherin-like and PC-esterase domain containing 1	**-5.6**	0.93595	0.013224
1384035_at	***Ildr2***	immunoglobulin-like domain containing receptor 2	**3.2**	0.742008	0.001312		1383117_at	***Pxmp4***	peroxisomal membrane protein 4	**-3.7**	0.93595	0.041268
1368290_at	***Cyr61***	cysteine-rich, angiogenic inducer, 61	**3.0**	0.93595	0.044878		1387319_at	***Ccl11***	chemokine (C-C motif) ligand 11	**-3.3**	0.697424	0.001068
1367631_at	***Ctgf***	connective tissue growth factor	**2.5**	0.93595	0.004885		1376711_at	***Cldn11***	claudin 11	**-3.0**	0.20371	1.97E-05
1374353_x_at	***Acta2 /// Actc1***	actin, alpha 2, smooth muscle, aorta /// actin, alpha, cardiac muscle 1	**2.5**	0.500296	0.000197		1368990_at	***Cyp1b1***	cytochrome P450, family 1, subfamily b, polypeptide 1	**-3.0**	0.93595	0.00797
1380866_at	***LOC365985***	similar to adenylate kinase 5 isoform 1	**2.1**	0.93595	0.026317		1397684_at	***Dkk2***	dickkopf WNT signaling pathway inhibitor 2	**-2.7**	0.93595	0.011909
1387410_at	***Nr4a2***	nuclear receptor subfamily 4, group A, member 2	**2.0**	0.93595	0.046111		1388018_at	***Sele***	selectin E	**-2.6**	0.93595	0.00805
1368146_at	***Dusp1***	dual specificity phosphatase 1	**2.0**	0.93595	0.027788		1393421_at	***Pxmp4***	peroxisomal membrane protein 4	**-2.6**	0.93595	0.037052
1370405_at	***Mcpt1l1***	mast cell protease 1-like 1	**2.0**	0.93595	0.026427		1393645_at	***Mageb16***	melanoma antigen family B, 16	**-2.5**	0.93595	0.022558
1388056_at	***Oas1b***	2-5 oligoadenylate synthetase 1B	**1.9**	0.93595	0.027587		1377086_at	***C1qtnf3***	C1q and tumor necrosis factor related protein 3	**-2.5**	0.627551	0.00044875

Supplementary Table 4: The ten most altered transcripts (by fold change) in the urinary bladder of sucrose-treated rats compared to diabetic rats
Transcripts (with known genes) were sorted according to fold-change ($p<0.05$)

Sucrose-treated vs Diabetic												
**Increased**							**Decreased**					
**Probeset**	**Gene Symbol**	**Gene Title**	**Fold-change**	**fdr**	**p value**		**Probeset**	**Gene Symbol**	**Gene Title**	**Fold-change**	**fdr**	**p value**
1388202_at	***RT1-EC2***	RT1 class Ib, locus EC2	**6.4**	0.418079	0.020582		1392074_at	***Cped1***	cadherin-like and PC-esterase domain containing 1	**-7.1**	0.227515	0.003579
1370059_at	***Nefl***	neurofilament, light polypeptide	**4.6**	0.024615	1.35E-05		1369113_at	***Grem1***	gremlin 1	**-6.7**	0.136106	0.000727
1370269_at	***Cyp1a1***	cytochrome P450, family 1, subfamily a, polypeptide 1	**4.2**	0.470809	0.029116		1374683_at	***Sgcg***	sarcoglycan, gamma (dystrophin-associated glycoprotein)	**-6.0**	0.005972	5.76E-07
1368290_at	***Cyr61***	cysteine-rich, angiogenic inducer, 61	**4.1**	0.298173	0.007669		1371065_at	***LOC688090 /// RT1-Bb***	similar to RT1 class II histocompatibility antigen, B-1 beta chain precursor (RT1.B-bet	**-5.8**	0.585529	0.064054
1369217_at	***Nr4a3***	nuclear receptor subfamily 4, group A, member 3	**3.8**	0.300847	0.007888		1389408_at	***LOC100359539 /// Rrm2***	ribonucleotide reductase M2 polypeptide /// ribonucleotide reductase M2	**-5.3**	0.026228	2.19E-05
1370355_at	***Scd1***	stearoyl-Coenzyme A desaturase 1	**3.7**	0.055419	0.000122		1372685_at	***Cdkn3***	cyclin-dependent kinase inhibitor 3	**-4.5**	0.055419	9.80E-05
1388203_x_at	***RT1-CE10 /// RT1-EC2***	RT1 class I, locus CE10 /// RT1 class Ib, locus EC2	**3.7**	0.453545	0.026499		1392053_at	***Mmrn1***	multimerin 1	**-3.9**	0.024615	1.13E-05
1370150_a_at	***Thrsp***	thyroid hormone responsive	**3.1**	0.207612	0.002737		1381504_at	***Aspn***	asporin	**-3.8**	0.04268	4.80E-05
1369268_at	***Atf3***	activating transcription factor 3	**3.1**	0.491024	0.03272		1368224_at	***Serpina3n***	serine (or cysteine) peptidase inhibitor, clade A, member 3N	**-3.6**	0.229877	0.00374
1373759_at	***Fosb***	FBJ osteosarcoma oncogene B	**2.9**	0.431716	0.022697		1376799_a_at	***Crlf1***	cytokine receptor-like factor 1	**-3.4**	0.003826	1.23E-07

Supplementary Table 5. Indices of Diabetes. At 16 weeks, *db/db* mice were significantly heavier than their lean controls, hyperglycaemic and polyuric. Statistical analysis was conducted using an unpaired t-test or Mann-Whitney test as appropriate for the dataset (**** $p<0.0001$)

**Experimental details**	**Terminal measurements (16 weeks)**		
**Group** *(n number)*	**End weight** (g)	**Albuminuria (µg/16hr)**	**Blood glucose** (mg/dl)
db/lean **mice** *(16)*	30.3 ± 2.0	22.9 ± 10.0	145.8 ± 21.1
db/db **mice** *(16)*	50.4 ± 4.5****	86.4 ± 58.0****	605.2 ± 93.6****

Supplementary Figure 1.

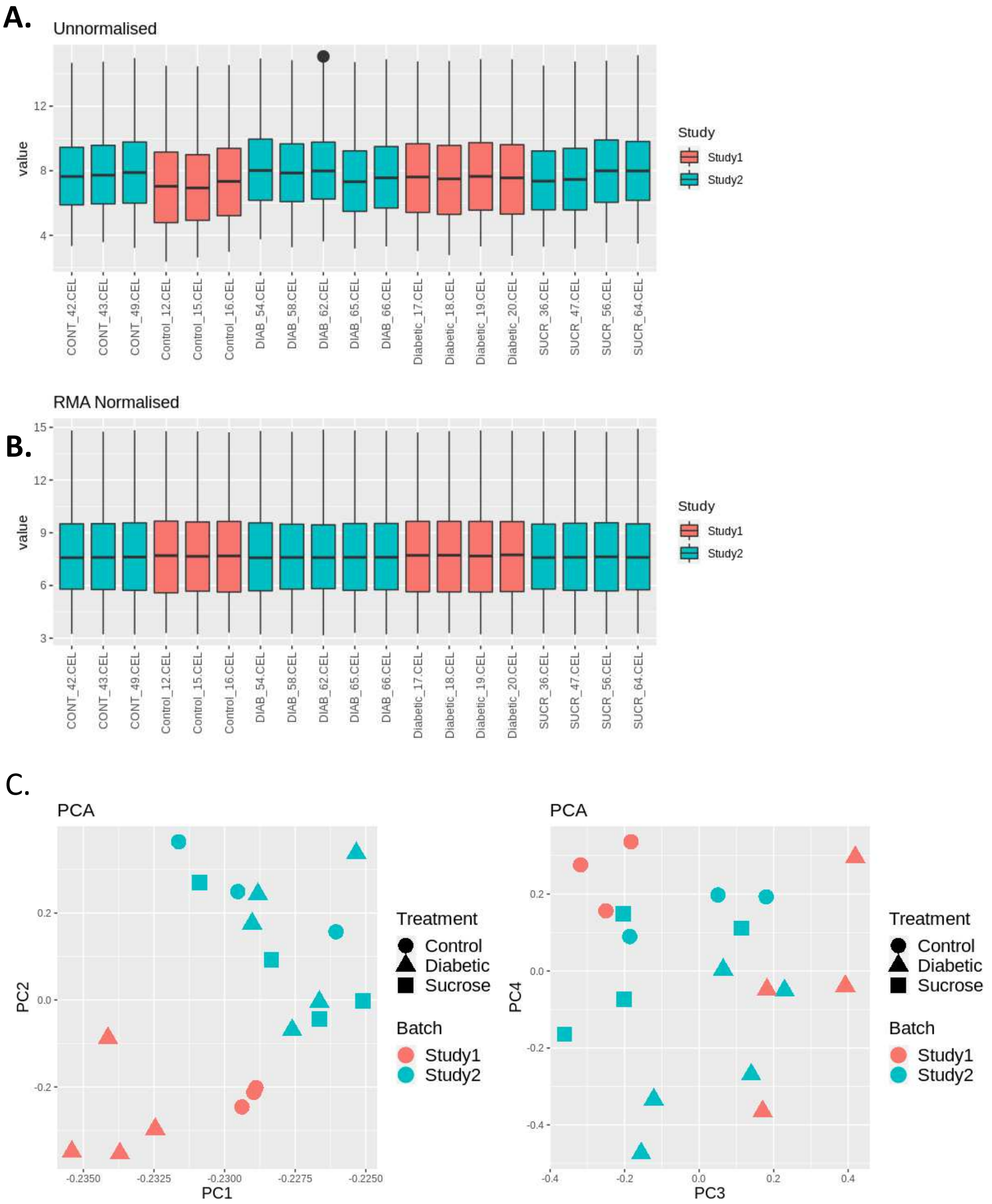

Supplementary Figure 1. Gene normalisation and Principal Component analysis plots for Affymetrix arrays. Gene expression analysis quantile normalisation, and background correction were conducted using RMA in Bioconductor and differential expression was done with limma in Bioconductor (PMID: 15461798). Boxplots show gene expression before **(A),** and after normalisation (**B**). Gene lists of differentially expressed genes were controlled for false discovery rate (fdr) errors using the method of QVALUE [24]. (**C**) Principal component analysis (PCA) was performed with Partek Genomics Suite (Partek Inc., USA.), Study 1: control *n=3*; diabetic *n=4;* Study 2: control *n=3*, diabetic *n=5* and sucrose-treated *n=4*.

Supplementary Figure 2.

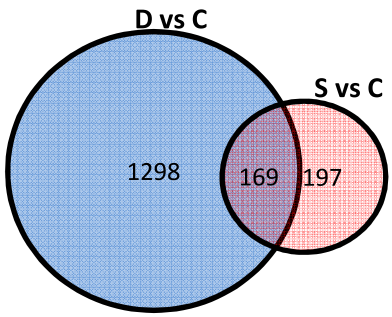

Supplementary Figure 2 GeneVenn analysis of significant transcriptomic changes in diabetic and sucrose-treated rats compared with controls. Differential gene expression was filtered by *P* value<0.05, a fold change > ± 1.3, transcripts with low threshold expression (<50) in all groups were excluded from analysis. Dysregulation of only 169 transcripts were common to both diabetic and sucrose-treated rats compared with control, highlighting the greater impact of DM, than polyuria alone on the bladder transcriptome after 16 weeks.

Supplementary Figure 3.

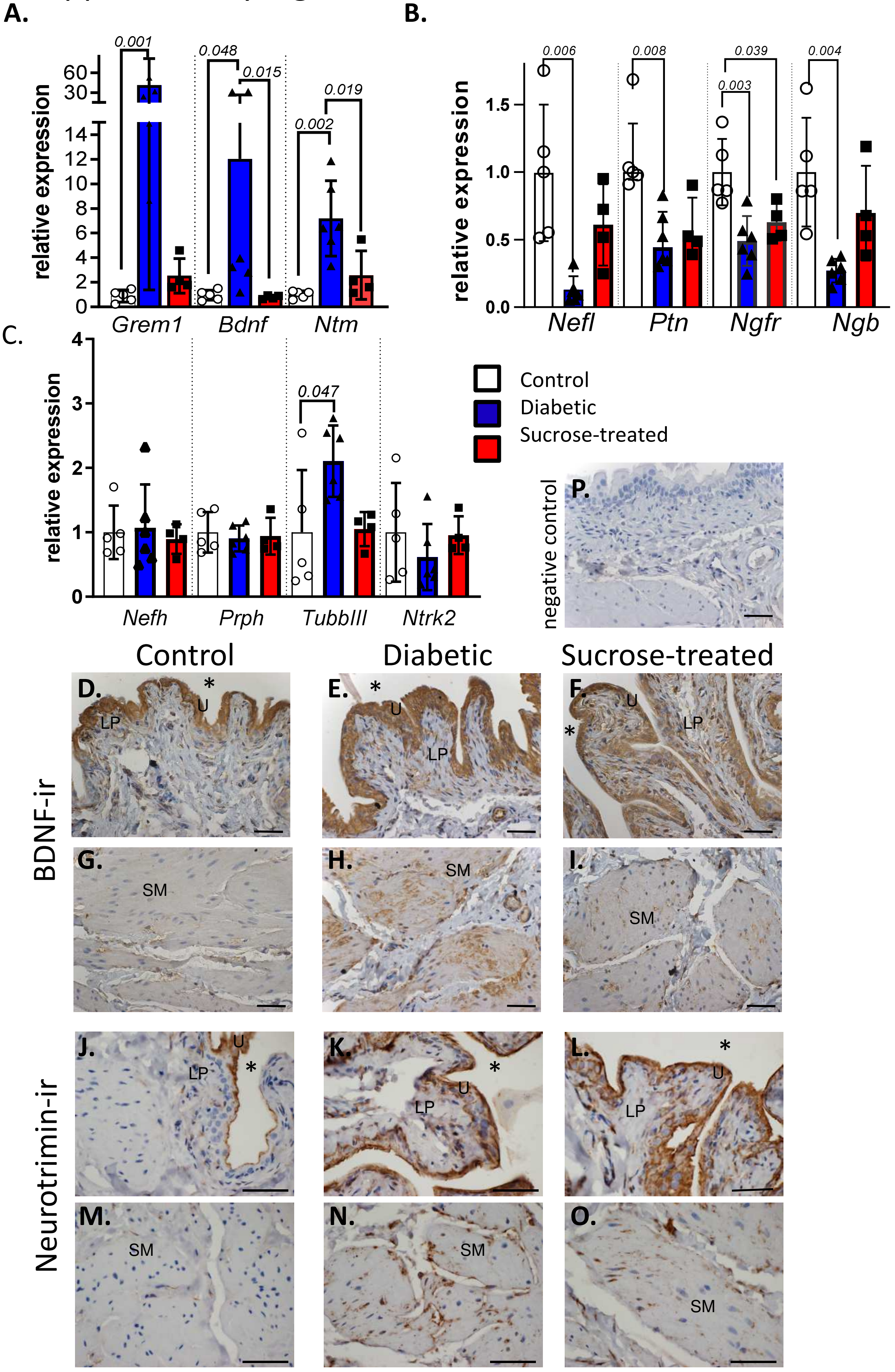

Supplementary Figure 3. Validation of array. Relative expression of selected transcripts (**A**) upregulated in array (diabetic versus control comparison: *p and fdr*$<$*0.05*) (**B**) downregulated in array (diabetic versus control comparison: *p and fdr*$<$*0.05*) and (**C**) not significantly altered in array (*p and fdr*$>$*0.05)* compared to control levels shows good reproducibility (control *n=5*, diabetic *n=6*; sucrose-treated *n=4*; $p<0.05$, one way ANOVA followed by Tukey's posthoc test, or Kruskal-Wallis and Dunns multiple comparison test, as appropriate for data). (**D-I**) Brain-derived neurotrophic factor (BDNF)-immunoreactivity (-ir: Abcam AB108319; 1:200) and (**J-O**) Neurotrimin-ir (EMD Millipore, AB2280; 1;250) were both detected in the urothelium (U) of control, DM and sucrose-treated rats. Signals for the two proteins were prominent in the detrusor smooth muscle (SM) layer of diabetic rats but appeared minimal in bladder SM of control rats. Sucrose-treated rats also showed –ir for both BDNF and neurotrimin in the bladder SM but these signals appeared less prominent than those in the diabetic rats. (**P)** negative control (minus primary antibody, diabetic rat tissue section). Scale bar = 50µm; * = bladder lumen; LP = lamina propria.

Supplementary Figure 4.

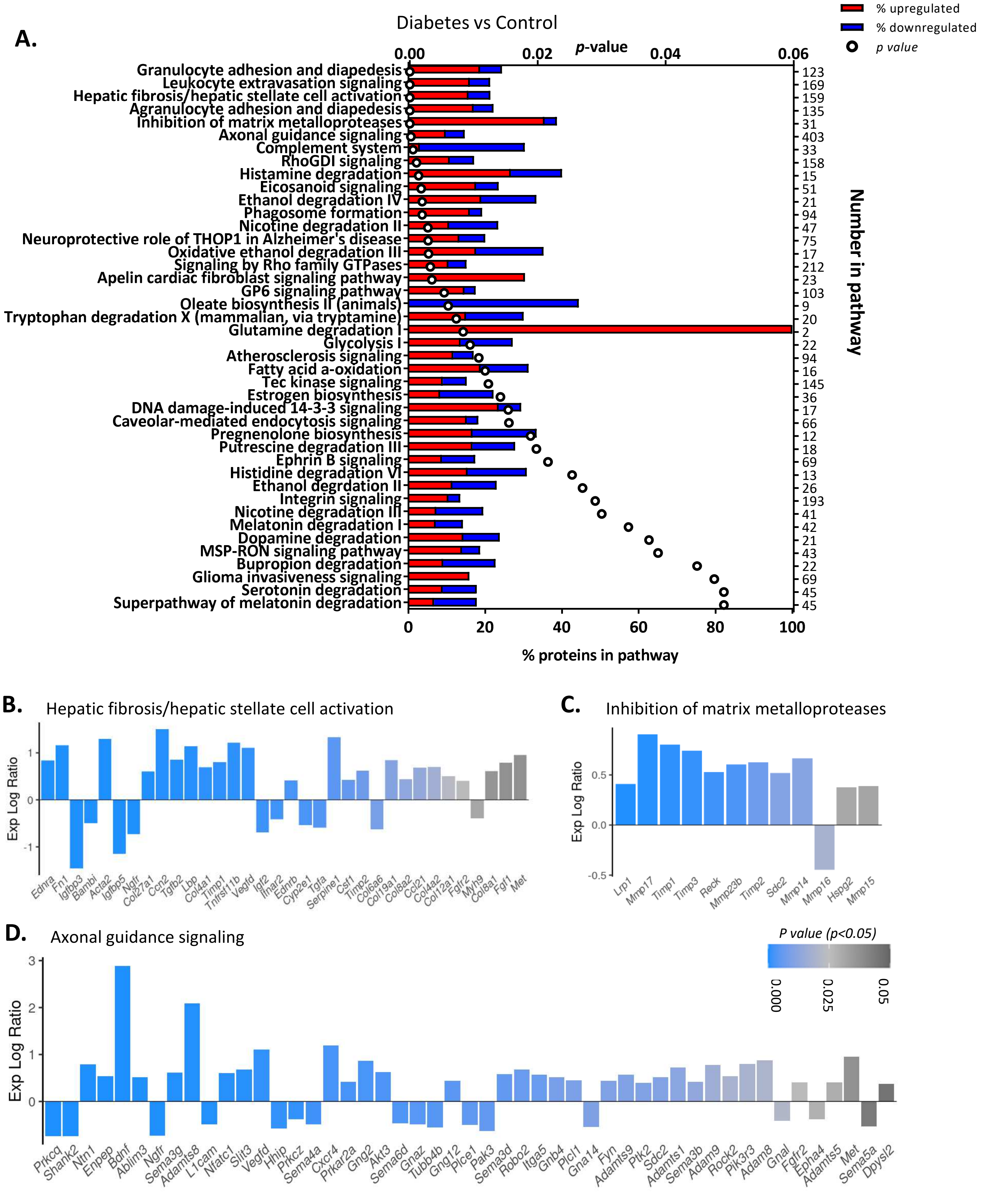

Supplementary Figure 4. Transcriptomic pathway analysis revealed 42 overrepresented pathways in the urinary bladder of diabetic compared to control rats. (**A**) Ingenuity Pathway Analysis characterised the overrepresented canonical pathways, organised by pathway names (left y-axis) and p-value (top x-axis); the bars show the % of differentially expressed transcripts (bottom x-axis; Red: upregulated; Blue: downregulated) and total number of molecules ascribed in each canonical pathway are shown on right-y-axis. The changes in expression of deregulated transcripts in the altered (**B**) '*Hepatic fibrosis/hepatic stellate cell activation*', (C) '*Inhibition of matrix metalloproteases*' and (D) '*Axonal guidance signalling*' pathways are arranged in order of significance (all $p<0.05$).

Supplementary Figure 5.

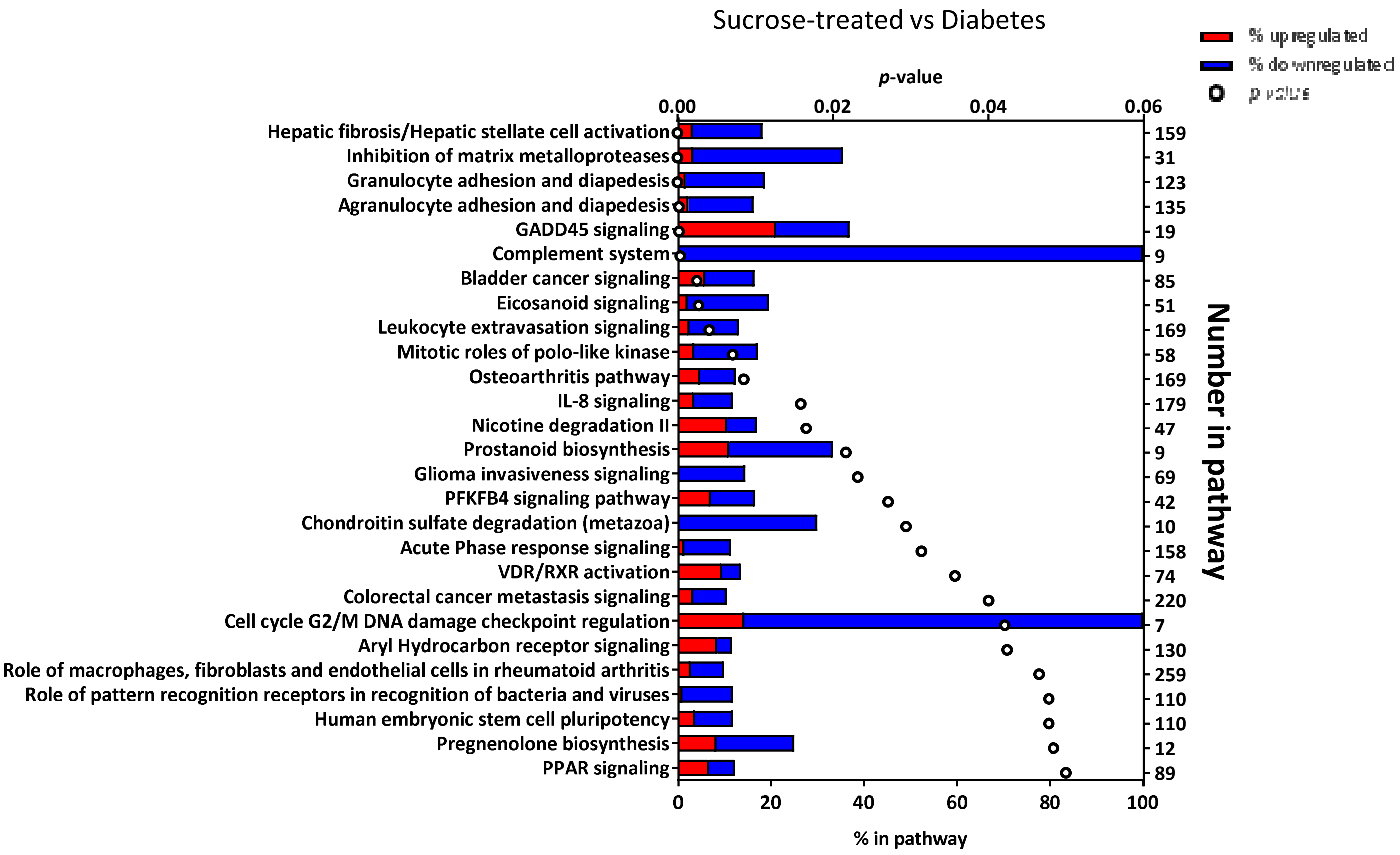

Supplementary Figure 5. Transcriptomic pathway analysis revealed 27 overrepresented pathways in the urinary bladder of sucrose-treated compared to control rats. Ingenuity Pathway Analysis characterised the overrepresented canonical pathways, organised by pathway names (left y-axis) and p-value (top x-axis); the bars show the % of differentially expressed transcripts (bottom x-axis; Red: upregulated; Blue: downregulated) and total number of molecules ascribed in each canonical pathway are shown on right-y-axis.

Supplementary Figure 6.

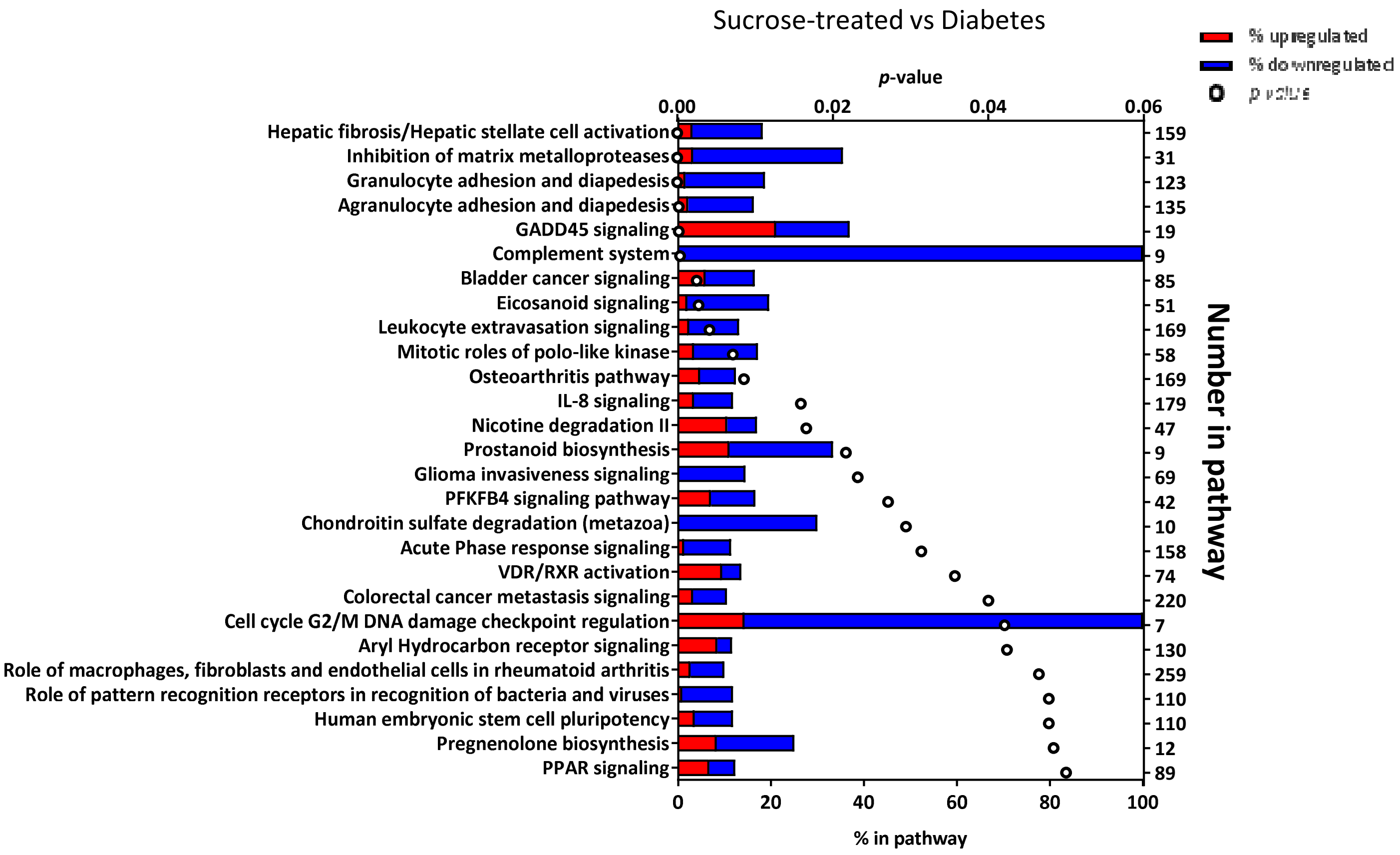

B. Hepatic fibrosis/hepatic stellate cell activation

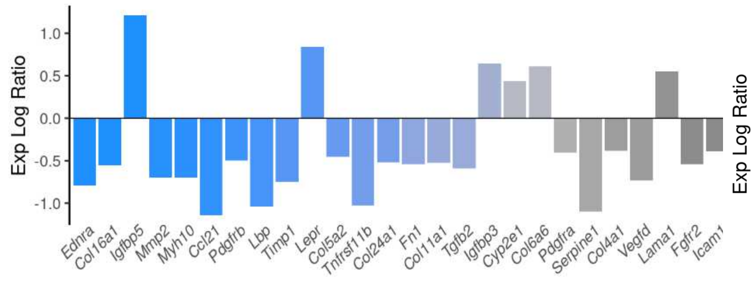

C. Inhibition of matrix metalloproteases

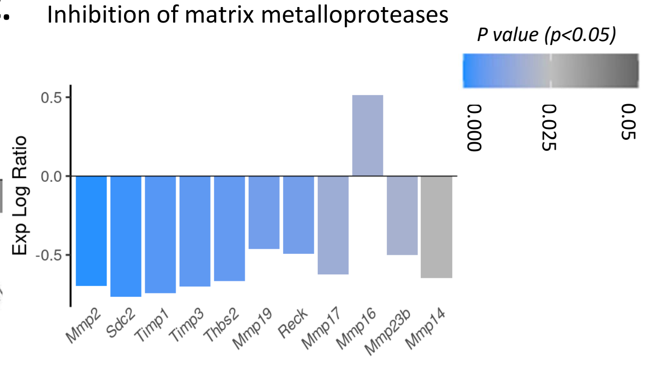

Supplementary Figure 6. Transcriptomic pathway analysis revealed 27 overrepresented pathways in the urinary bladder of sucrose-treated compared to diabetic rats. Ingenuity Pathway Analysis characterised the overrepresented canonical pathways, organised by pathway names (left y-axis) and p-value (top x-axis); the bars show the % of differentially expressed transcripts (bottom x-axis; Red: upregulated; Blue: downregulated) and total number of molecules ascribed in each canonical pathway are shown on right-y-axis. The changes in expression of deregulated transcripts in the altered (**B**) '*Hepatic fibrosis/hepatic stellate cell activation*', (C) '*Inhibition of matrix metalloproteases'* pathways are arranged in order of significance (all $p<0.05$).

Supplementary Figure 7

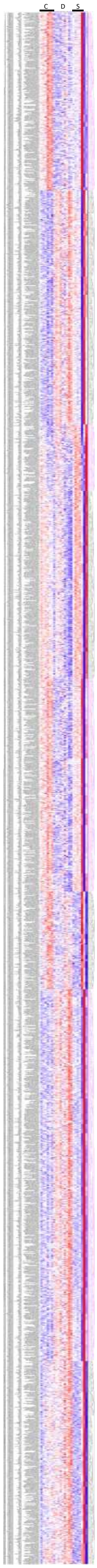

Supplementary Figure 7. Full cluster analysis demonstrates altered pattern of transcript expression in the bladder of control, diabetic and sucrose-treated rats. Unsupervised hierarchical clustering grouped transcripts into eight clusters. Rows are the mean transcript expression levels denoted as the z-score displayed in colourised (High (Red) to Low (Blue)) scale. Columns are data from individual animals (Control *n=6*; Diabetic *n=9*; Sucrose-treated *n=4*), means are expressed in the right-hand columns. Refer to separate supplementary .png file.

Supplementary Figure 8.

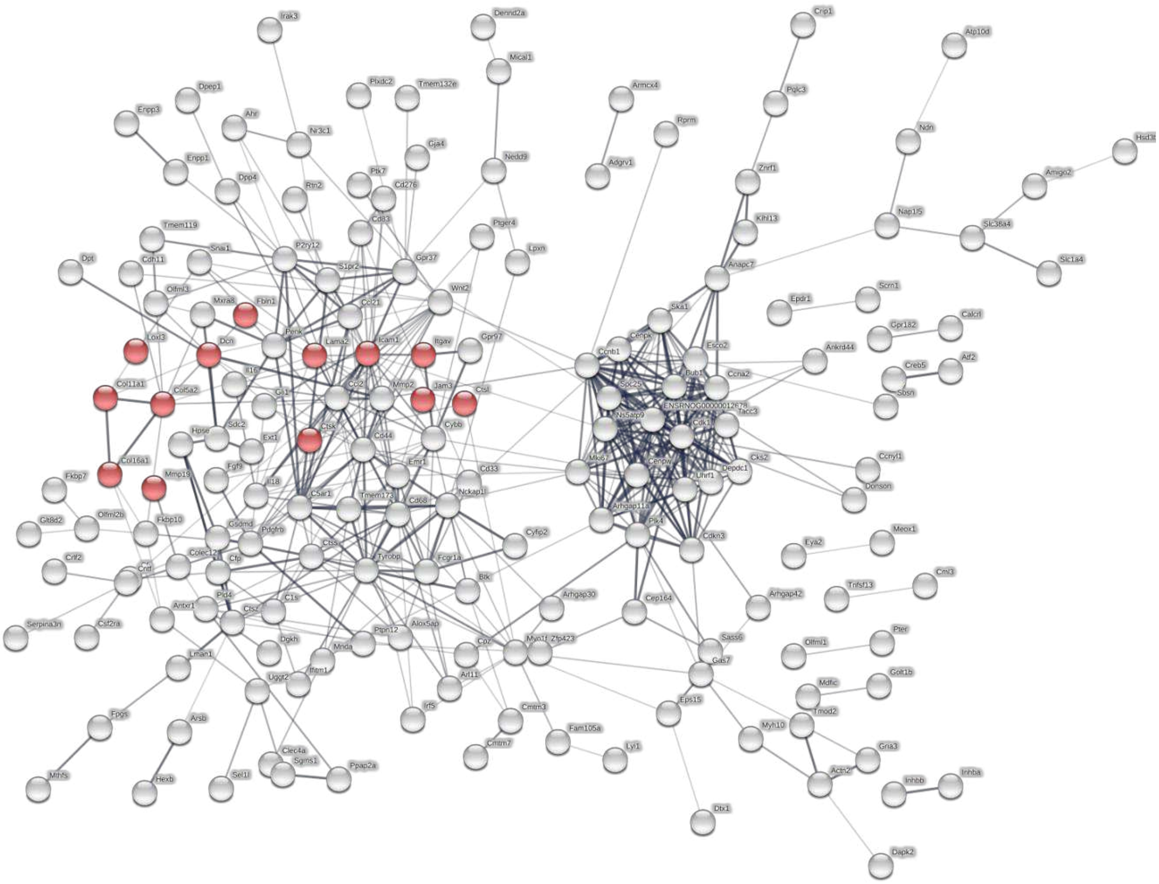

Supplementary Figure 8. STRING network analysis of Cluster 8. Interaction networks of the connected annotated molecules in Cluster 8. The most significant reactome pathway (‘*extracellular matrix organisation’)* are highlighted as red coloured nodes.

Supplementary Figure 9.

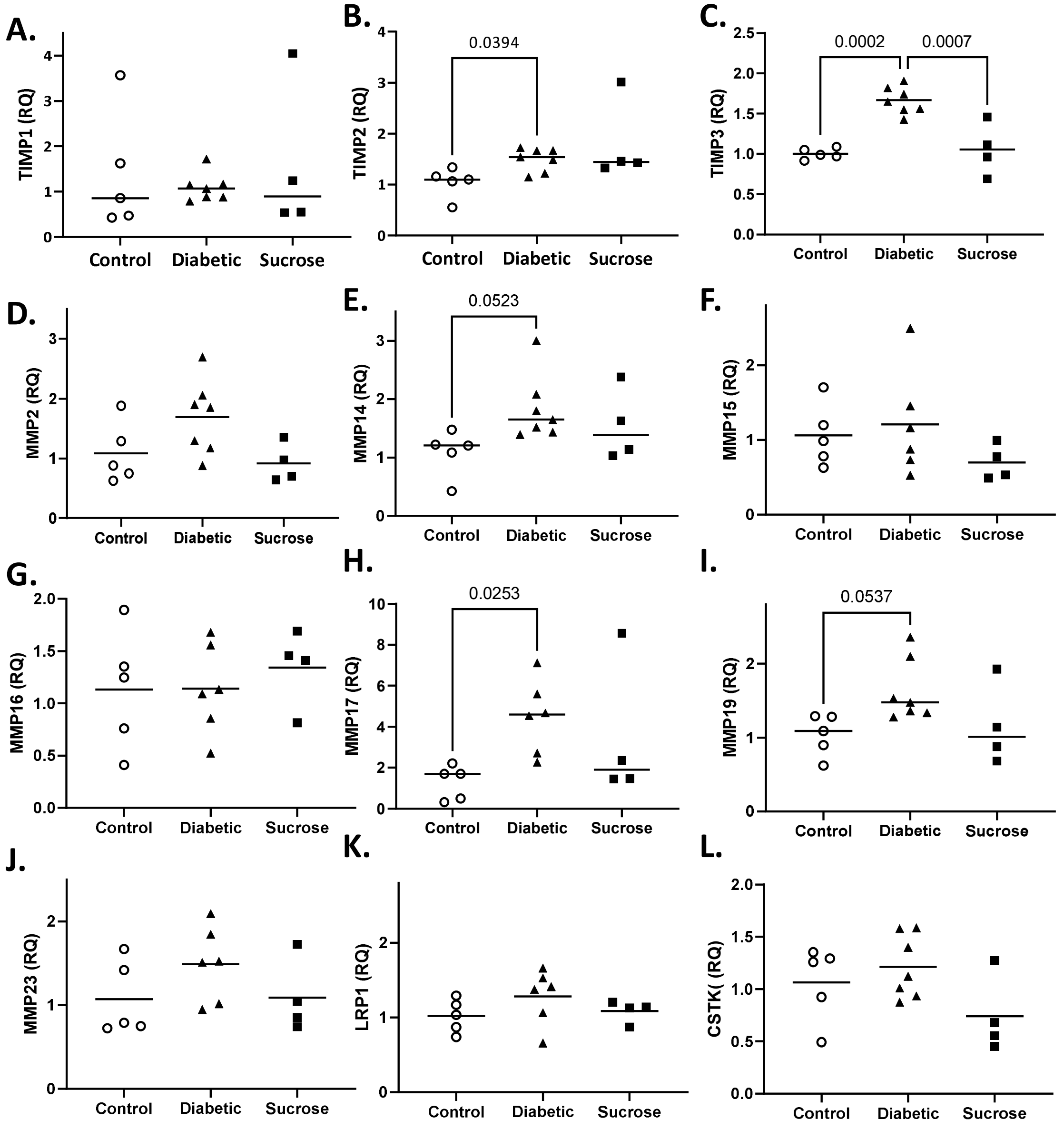

Supplementary Figure 9. qRT-PCR on genes from *'Inhibition of matrix metalloproteinases'* pathway IPA pathway and Cluster 8. Relative expression of **(A)** *Timp1,* **(B)** *Timp2***, (C)** *Timp3* **(D)** *Mmp2,* **(E)** *Mmp14,* **(F)** *Mmp15,* **(G)** *Mmp16,* **(H***) Mmp17,* **(I)** *Mmp19,* **(J)** *Mmp23,* **(K***) Lrp1 and* **(L)** *Ctsk*, normalised to Actb and expressed relative to control group expression. Data are expressed as mean ± SD or median ± IQR and analysed by one way ANOVA followed by Tukey's posthoc test, or Kruskal-Wallis and Dunns multiple comparison test, as appropriate for data sets.

Supplementary Figure 10.

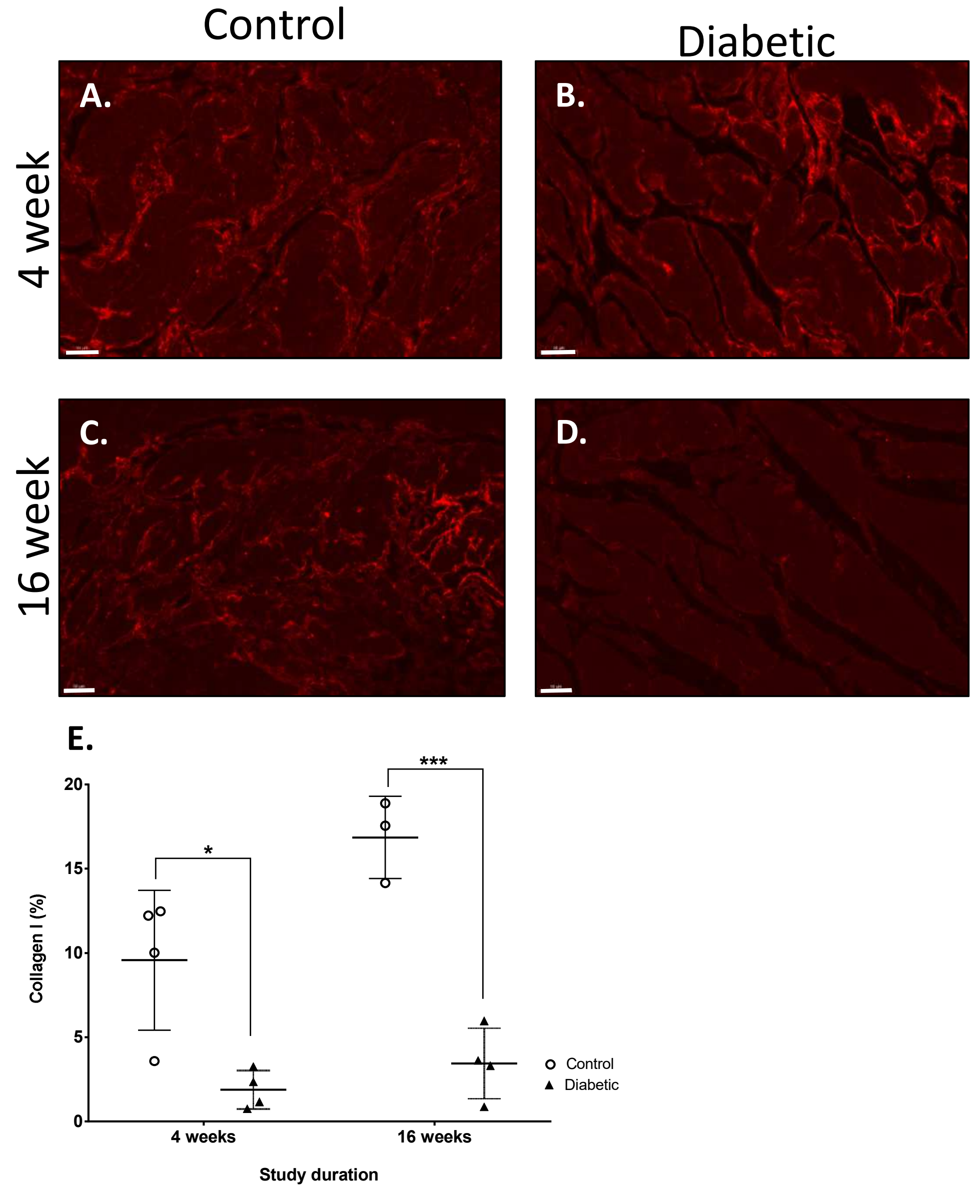

Supplementary Figure 10. Collagen I-immunoreactivity is downregulated in the detrusor muscle of diabetic rats. Urinary bladder transverse sections were immunostained for collagen I. Bladders from control (**A, C**) rats showed collagen I-immunoreactivity around muscle bundles in the detrusor muscle. Collagen I-immunoreactivity decreased in the detrusor muscle (**E**) of rats after 4 (**B**) and 16 weeks (**D**) of diabetes. Scale bar = 100 μm. Data (**E**) are expressed as mean ± SD, analysed by t-test ($^{*}p<0.05$, *** $p<0.001$).

Supplementary Figure 11.

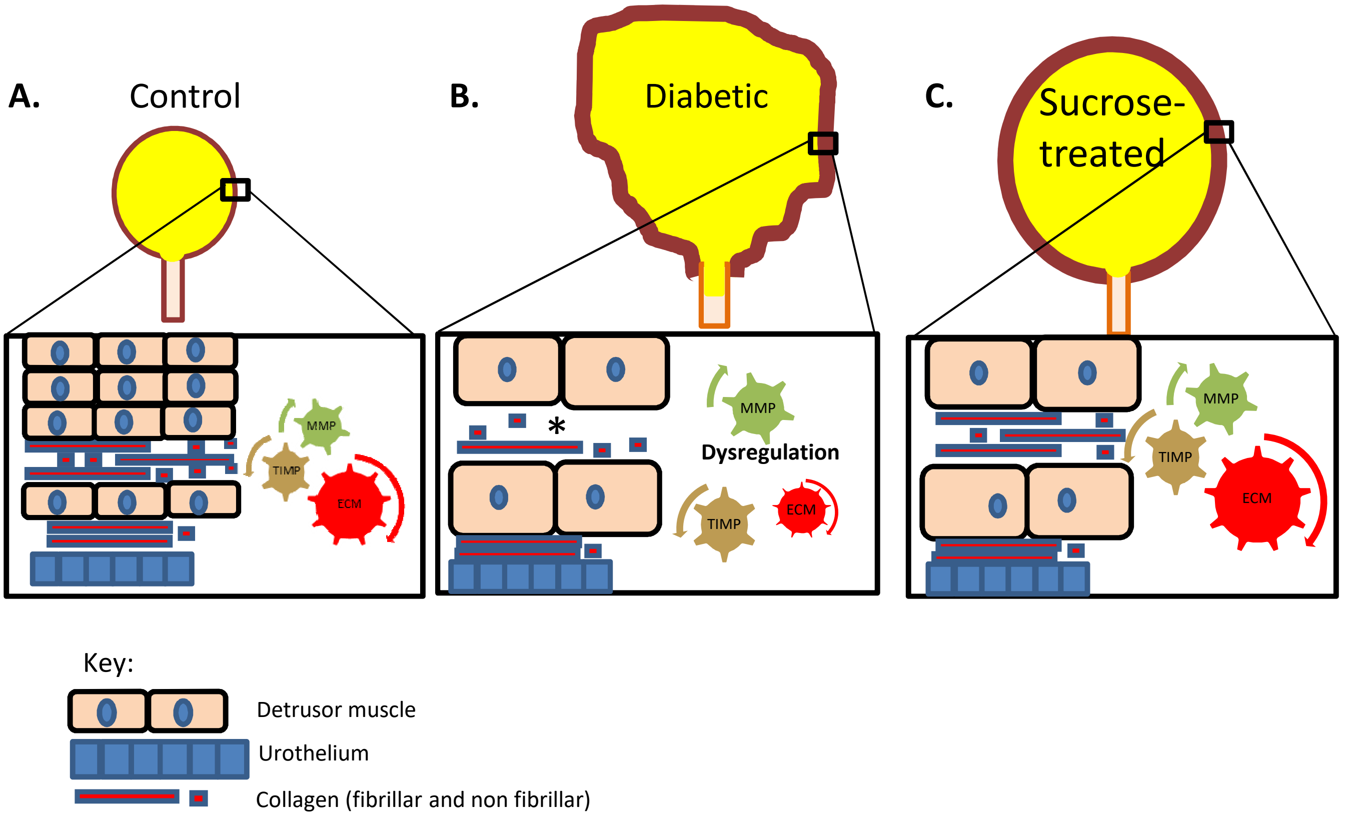

Supplementary Figure 11. Cartoon schematic of bladder wall changes occurring in DM and polyuria alone. Detrusor muscle hypertrophy occurred similarly in models of STZ-induced DM and rats with polyuria, with an increase in wall-thickness and bladder size. We found specific molecular changes occur in the urinary bladder in experimental DM. Gene array and bioinformatics highlighted dysregulation of ECM regulatory pathways and we found a loss of birefringent collagen fibrils (*) in the detrusor muscle of diabetic rats. AFM revealed a reduction in tissue stiffness in detrusor muscle of rats with DM, compared with control and sucrose-treated rats. Remodelling of the bladder wall, with the DM-associated changes in ECM homeostasis may contribute towards the reduced tissue rigidity. We suggest this may be central to causing the incomplete voiding reported in people who have had long-term bladder damage associated with diabetes mellitus.
